# Elevated endogenous GDNF induces altered dopamine signalling in mice and correlates with clinical severity in schizophrenia

**DOI:** 10.1038/s41380-022-01554-2

**Published:** 2022-05-26

**Authors:** Kärt Mätlik, Daniel R. Garton, Ana R. Montaño-Rodríguez, Soophie Olfat, Feride Eren, Laoise Casserly, Anastasios Damdimopoulos, Anne Panhelainen, L. Lauriina Porokuokka, Jaakko J. Kopra, Giorgio Turconi, Nadine Schweizer, Erika Bereczki, Fredrik Piehl, Göran Engberg, Simon Cervenka, T. Petteri Piepponen, Fu-Ping Zhang, Petra Sipilä, Johan Jakobsson, Carl M. Sellgren, Sophie Erhardt, Jaan-Olle Andressoo

**Affiliations:** 1 Department of Pharmacology, Faculty of Medicine, Neuroscience Center & Helsinki Institute of Life Science, University of Helsinki, 00290 Helsinki, Finland; 2 Division of Neurogeriatrics, Department of Neurobiology, Care Sciences and Society (NVS), Karolinska Institutet, 14183 Huddinge, Sweden; 3 Department of Physiology and Pharmacology, Karolinska Institutet, 17177 Stockholm, Sweden; 4 Department of Biosciences and Nutrition, Karolinska Institutet, 14183 Huddinge, Sweden; 5 Institute of Biotechnology, University of Helsinki, 00014 Helsinki, Finland; 6 Division of Pharmacology and Pharmacotherapy, Faculty of Pharmacy, University of Helsinki, 00014 Helsinki, Finland; 7 Department of Clinical Neuroscience, Neuroimmunology Unit, Karolinska Institutet, Karolinska University Hospital, 17177 Stockholm, Sweden; 8 Centre for Psychiatry Research, Department of Clinical Neuroscience, Karolinska Institutet & Stockholm Health Care Services, Region Stockholm, 17177 Stockholm, Sweden; 9 Department of Medical Sciences, Psychiatry, Uppsala University, 75185 Uppsala, Sweden; 10 Research Centre for Integrative Physiology and Pharmacology, Institute of Biomedicine and Turku Center for Disease Modeling, University of Turku, 20520 Turku, Finland; 11 GM-Unit, Laboratory Animal Center, Helsinki Institute of Life Science, University of Helsinki, 00290 Helsinki, Finland; 12 Laboratory of Molecular Neurogenetics, Department of Experimental Medical Science, Wallenberg Neuroscience Center and Lund Stem Cell Center, BMC A11, Lund University, 221 84 Lund, Sweden

**Keywords:** Schizophrenia, Neuroscience, Biological techniques

## Abstract

Presynaptic increase in striatal dopamine is the primary dopaminergic abnormality in schizophrenia, but the underlying mechanisms are not understood. Here, we hypothesized that increased expression of endogenous GDNF could induce dopaminergic abnormalities that resemble those seen in schizophrenia. To test the impact of GDNF elevation, without inducing adverse effects caused by ectopic overexpression, we developed a novel in vivo approach to conditionally increase endogenous GDNF expression. We found that a 2–3-fold increase in endogenous GDNF in the brain was sufficient to induce molecular, cellular, and functional changes in dopamine signalling in the striatum and prefrontal cortex, including increased striatal presynaptic dopamine levels and reduction of dopamine in prefrontal cortex. Mechanistically, we identified adenosine A2a receptor (A_2A_R), a G-protein coupled receptor that modulates dopaminergic signalling, as a possible mediator of GDNF-driven dopaminergic abnormalities. We further showed that pharmacological inhibition of A_2A_R with istradefylline partially normalised striatal GDNF and striatal and cortical dopamine levels in mice. Lastly, we found that GDNF levels are increased in the cerebrospinal fluid of first episode psychosis patients, and in post-mortem striatum of schizophrenia patients. Our results reveal a possible contributor for increased striatal dopamine signalling in a subgroup of schizophrenia patients and suggest that GDNF—A_2A_R crosstalk may regulate dopamine function in a therapeutically targetable manner.

## Introduction

Schizophrenia is a debilitating neuropsychiatric disease affecting 1% of the global population. The clinical features of schizophrenia include positive, negative, and cognitive symptoms, and at least some of these clinical manifestations are thought to reflect neurochemical disturbances, such as abnormalities in dopamine, glutamate, serotonin, adenosine, and gamma-aminobutyric acid signalling [1–5]. Among the various abnormalities observed in individuals with schizophrenia, dysfunctional dopamine signalling is a commonly reported pathology [6]. Recent studies suggest that in patients with disturbed dopamine signalling, increased presynaptic dopamine synthesis capacity and release in the nigrostriatal dopamine pathway are the primary dopaminergic abnormalities [7–10]. In addition, striatal hyperdopaminergia is associated with reduced dopamine function in the prefrontal cortex (PFC) [11–13]. However, there are currently no animal models showing that increased presynaptic function in the striatum can drive other schizophrenia-like phenotypes, including the cortical hypodopaminergia. As a result, we have limited understanding of the underlying mechanisms driving these abnormalities, and of possible treatment strategies.

Glial cell line-neurotrophic factor (GDNF) is a secreted protein expressed predominantly in striatal interneurons [14]. GDNF is a potent neurotrophic factor for midbrain dopamine neurons in culture [15] and promotes dopamine synthesis and dopaminergic neuron fibre outgrowth if applied ectopically into the brain in animal models and humans [16–20]. Furthermore, amphetamine, a drug that increases synaptic dopamine levels and is associated with increased susceptibility to developing schizophrenia [21–28], increases endogenous GDNF expression in the nigrostriatal tract [21, 24], and a two-fold increase in endogenous GDNF levels is sufficient to increase striatal dopamine synthesis and release in mice [29]. Despite these multiple lines of suggestive evidence, functional analysis linking GDNF signalling to schizophrenia is currently lacking.

We and others have shown that ectopic gene overexpression in highly structured tissues, including the brain, can induce side-effects and artefacts, yielding results that are difficult to interpret [29–40]. These findings have suggested that retaining the physiological gene expression pattern and levels is particularly important for studying the function of morphogens such as GDNF. We recently showed that Gdnf 3'UTR contains negative regulation elements and that the replacement of Gdnf 3'UTR with a 3'UTR less responsive to negative regulation enhances GDNF expression by about 2–3-fold at the post-transcriptional level, limited to cells that normally express GDNF [29].

Here, we generate a novel mouse model allowing us to conditionally upregulate endogenous GDNF. We find that increased levels of GDNF are sufficient to induce altered dopamine signalling, including striatal hyperdopaminergia and cortical hypodopaminergia, as well as behavioural deficits and gene expression alterations similar to individuals with schizophrenia. We then identify A_2A_R as a possible therapeutic target and show that administration of specific A_2A_R antagonist istradefylline partially normalises striatal and cortical dopamine levels in mice. Lastly, we find that GDNF levels are increased in the cerebrospinal fluid (CSF) of first episode psychosis (FEP) patients and in post-mortem striata of schizophrenia patients. Thus, our work demonstrates that 2–3-fold elevation in endogenous GDNF expression increases striatal presynaptic dopaminergic signalling and induces hypodopaminergia in prefrontal cortex in mice and suggests that elevated GDNF could contribute to the disease in a subgroup of patients with elevated GDNF levels. Our data also suggests A_2A_R as a potential drug target in a subgroup of schizophrenia patients with elevated GDNF.

## Results

### Generation of *Gdnf* conditional hypermorph allele

**Fig. 1:**
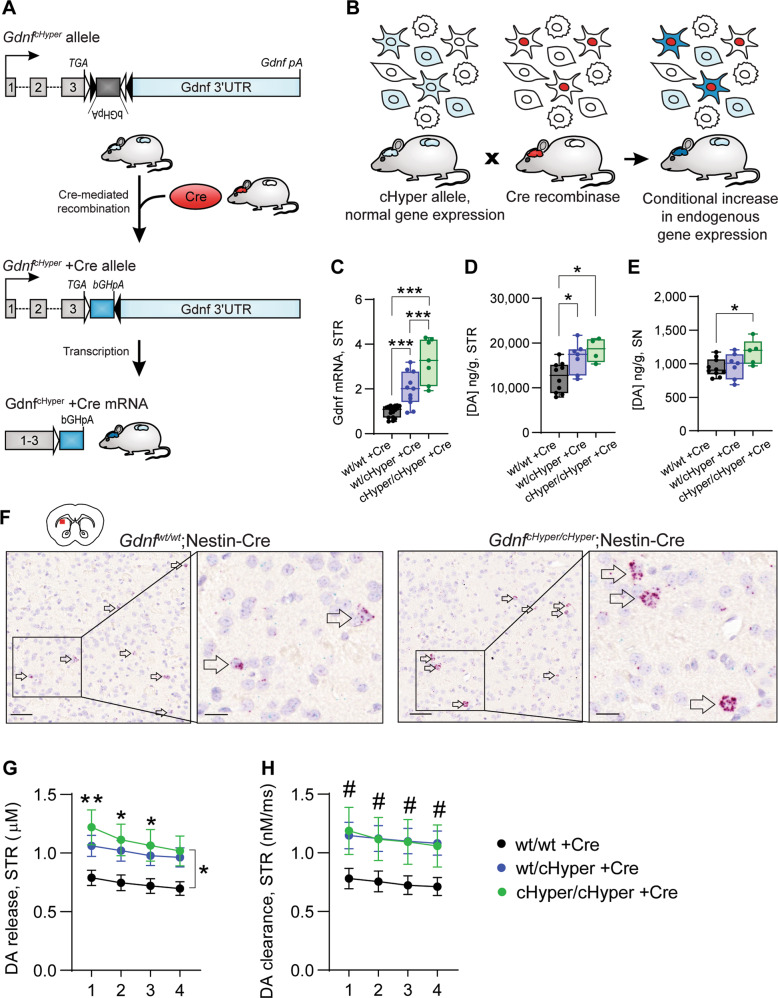
Increased endogenous GDNF levels increase striatal dopamine function. See also Supplementary Fig. S1. **A** Schematic of the *Gdnf* ^*cHyper*^ allele (top panel) and the resulting mRNA carrying a short 3'UTR (bottom panel) devoid of negative regulation elements in the 3'UTR. pA, polyadenylation signal. **B** Schematic of the conditional gene upregulation strategy to elevate endogenous gene expression in natively expressing cells. When using the conditional hypermorph allele, Cre-mediated recombination would result in an elevated gene expression specifically in cells that normally express the gene. **C** Striatal Gdnf mRNA levels in adult *Gdnf* ^*cHyper*^;Nestin-Cre mice, measured with qPCR. Box plots show median, upper, and lower quartiles, as well as maximum and minimum values. *N* = 7–17 mice per group. One-way ANOVA, Tukey’s multiple comparisons test. ***p < 0.001. **D** Total tissue dopamine levels in the striatum, measured with HPLC. Box plots show median, upper, and lower quartiles, as well as maximum and minimum values. *N* = 4–10 mice per group. One-way ANOVA, Tukey’s multiple comparisons test. *p < 0.05. **E** Total tissue dopamine levels in the substantia nigra, measured with HPLC. Box plots show median, upper, and lower quartiles, as well as maximum and minimum values. *N* = 5–10 mice per group. One-way ANOVA, Tukey’s multiple comparisons test. *p < 0.05. **F** Gdnf mRNA expression pattern in dorsal striatum, detected using RNAscope probes. Arrows indicate cells with high Gdnf signal. Scale bar 50 µm (**d**, left panels) or 10 µm (**d**, right panels, higher magnification). **G** Stimulated dopamine release in striatal slices across four consecutive stimulations, measured with fast-scan cyclic voltammetry. Mean ± SEM. *n* = 10–20 striatal slices (from *N* = 4–8 mice) per group. Two-way repeated measures ANOVA, Tukey’s multiple comparisons test. *p < 0.05; **p < 0.01 between cHyper/cHyper +Cre and wt/wt +Cre. **H** Similar to (**G**) but showing dopamine re-uptake. Mean ± SEM. *n* = 10–20 striatal slices (from *N* = 4–8 mice) per group. Two-way repeated measures ANOVA, Tukey’s multiple comparisons test. ^#^*p* < 0.05 between wt/cHyper +Cre and wt/wt +Cre.

GDNF is a neurotrophic factor expressed in striatal interneurons that stimulate dopamine signalling in the nigrostriatal dopamine pathway [14–16, 20, 29]. At the same time, increased dopamine synthesis capacity and release in dorsal striatum have been associated with psychosis in schizophrenia patients [8]. We asked whether elevated endogenous GDNF expression in mice is sufficient to induce striatal hyperdopaminergia and other schizophrenia-associated changes in the brain. To avoid ectopic GDNF overexpression related developmental defects [29, 41–45], we generated a novel conditional hypermorph allele enabling an increase in the endogenous, spatially unaltered expression of GDNF in a temporally controlled manner using the Cre-Lox system (*Gdnf* conditional hypermorphic mice, *Gdnf* ^*cHyper*^). To that end, we inserted a FLEx cassette [46] containing the bovine growth hormone polyadenylation sequence (bGHpA; a 3'UTR sequence previously shown to lead to increased expression from the cognate mRNA [47]) in an inverted orientation immediately downstream of the stop codon of the mouse *Gdnf* gene (Fig. 1A and Supplementary Fig. S1A, B). Based on our previous work on Gdnf 3'UTR [29], we hypothesised that by affecting only post-transcriptional regulation, Cre-mediated recombination would result in increased *Gdnf* expression without changing *Gdnf*’s expression pattern (Fig. 1B).

To verify endogenous GDNF overexpression in *Gdnf* ^*cHyper*^ mice, we first analysed the kidneys, where *Gdnf* expression pattern and the outcome of *Gdnf* gene level alterations are well characterised. Mice lacking GDNF have no kidneys [41–43], whereas constitutive endogenous GDNF overexpression causes a reduction in kidney size and other histological alterations [29, 44, 45]. In line with these studies, crossing *Gdnf* ^*cHyper*^ mice to a Pgk1-Cre line, where Cre expression results in ubiquitous recombination [48], resulted in increased Gdnf mRNA and GDNF protein expression and reduced kidney size (Supplementary Fig. S1C–E). Similarly, crossing *Gdnf* ^*cHyper*^ mice to a tamoxifen-inducible Esr1-Cre line [49] increased Gdnf mRNA expression in embryonic kidneys after tamoxifen administration to pregnant females, while retaining the developmental stage-appropriate expression pattern of *Gdnf* (Supplementary Fig. S1F, G). These results demonstrate that, as hypothesised, the *Gdnf* ^*cHyper*^ allele enables temporally controlled increase of endogenous *Gdnf* without altering its expression pattern.

### Increased endogenous GDNF levels increase striatal dopamine function

Next, we addressed how CNS-specific overexpression of GDNF affects striatal dopamine function. We crossed *Gdnf* ^*cHyper*^ mice to a Nestin-Cre line, where Cre is expressed specifically in the CNS during mid-gestation (E11.5 onwards) [50]. In the nigrostriatal system, GDNF is mostly produced by a sparse and regularly spaced population of striatal parvalbumin interneurons [14] that regulate striatal output [51]. We found that Gdnf mRNA and GDNF protein levels were increased in an allele dose-dependent manner in the striatum of 2-month-old *Gdnf* ^*cHyper*^;Nestin-Cre mice (Fig. 1C and Supplementary Fig. S1H), whereas Gdnf’s expression pattern remained normal (Fig. 1F). We then found that Nestin-Cre-driven elevation of endogenous GDNF significantly increased the levels of total tissue dopamine in the striatum and substantia nigra (SN) (Fig. 1D, E). In addition, fast-scan cyclic voltammetry (FSCV) experiments, measuring the kinetics of phasic dopamine release and re-uptake in acute brain slices, demonstrated increased dopamine release and re-uptake in the striatum (Fig. 1G, H). The tissue levels of dopamine metabolites 3,4-dihydroxyphenylacetic acid (DOPAC) and homovanillic acid (HVA) in the striatum and SN were not altered (Supplementary Fig. S1I, J), and the total number of dopaminergic neurons in the SN was normal (Supplementary Fig. S1K). Altogether, these results demonstrated that developmental upregulation of endogenous GDNF in *Gdnf* ^*cHyper*^;Nestin-Cre mice at mid-gestation results in increased striatal presynaptic dopamine function.

### Increased endogenous GDNF levels reduce dopamine signalling in the PFC

**Fig. 2:**
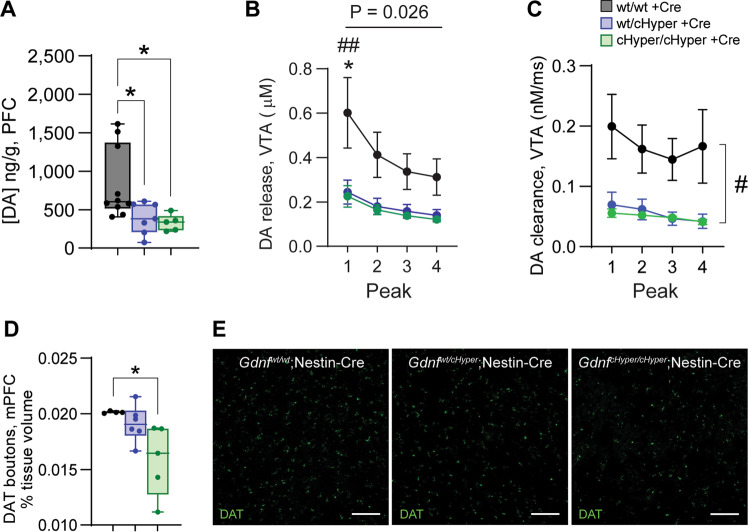
Increased endogenous GDNF levels reduce dopamine signalling in the PFC. See also Supplementary Fig. S2. **A** Total dopamine levels in the PFC, measured with HPLC. Box plots show median, upper, and lower quartiles, as well as maximum and minimum values. *N* = 5–10 mice per group. One-way ANOVA, Tukey’s multiple comparisons test. *p < 0.05. **B** Stimulated dopamine release in slices from ventral tegmental area (VTA) across four consecutive stimulations, measured with fast-scan cyclic voltammetry. Mean ± SEM. *n* = 2–6 striatal slices per group, one slice per animal. Two-way repeated measures ANOVA, Tukey’s multiple comparisons test. ^#^*p* < 0.05; ^##^*p* < 0.01 between *Gdnf* ^*wt/wt*^;Nestin-Cre and *Gdnf* ^*wt/cHyper*^;Nestin-Cre mice. **C** Similar to (**B**) but showing dopamine re-uptake. Mean ± SEM. *n* = 2–6 striatal slices per group, one slice per animal. Two-way repeated measures ANOVA, Tukey’s multiple comparisons test. ^#^*p* < 0.05 between *Gdnf* ^*wt/wt*^;Nestin-Cre and *Gdnf* ^*wt/cHyper*^;Nestin-Cre mice. **D** Quantification of the number of dopamine transporter (DAT)-positive synaptic boutons in the prelimbic area in the PFC. Box plots show median, upper, and lower quartiles, as well as maximum and minimum values. *N* = 4–6 mice per group (average values from *n* = 2–3 slices per animal). One-way ANOVA, Tukey’s multiple comparisons test. *p < 0.05. **E** Representative images of immunohistochemistry for DAT showing dopamine synapses in the prelimbic area in the PFC. Scale bar 50 µm.

Studies on schizophrenia patients suggest that increased striatal dopamine signalling associates with reduced dopamine signalling in the PFC [7, 12, 52–54]. Therefore, we asked if increased dopaminergic signalling in the striatum is associated with a cortical hypodopaminergia in *Gdnf* ^*cHyper*^;Nestin-Cre mice. We found a marked reduction in PFC dopamine levels in both *Gdnf* ^*wt/cHyper*^;Nestin-Cre and *Gdnf* ^*cHyper/cHyper*^;Nestin-Cre mice compared with controls (Fig. 2A), while the levels of DOPAC and HVA remained unchanged (Supplementary Fig. S2A, B). We next analysed dopamine signalling in the ventral tegmental area (VTA) using FSCV recordings in the VTA subregion that gives rise to the mesocortical dopamine fibres innervating the PFC [55]. We found a substantial decrease in somatodendritic dopamine release and reuptake (Fig. 2B, C), suggesting that dopaminergic signalling is reduced in the mesocortical pathway. Further analysis at the cellular level revealed a reduced number of dopamine transporter-positive (DAT+) dopaminergic synaptic boutons in the medial PFC and in the association cortex in *Gdnf* ^*cHyper/cHyper*^;Nestin-Cre mice compared with controls (Fig. 2D, E, Supplementary Fig. S2C, D), in line with reduced PFC dopaminergic innervation observed in post-mortem samples from individuals with schizophrenia [52]. At the same time, the number of dopamine neurons in the VTA was unchanged (Supplementary Fig. S2E), and the levels of dopamine, and dopamine metabolites DOPAC and HVA in ventral striatum showed a small, non-significant increase in *Gdnf* ^*cHyper/cHyper*^;Nestin-Cre mice compared with controls (Supplementary Fig. S2F–H). These results align with findings from humans, suggesting enhanced dopamine synthesis and release in dorsal but not in ventral striatum [8, 10, 56]. Altogether, these results demonstrate that an increase in presynaptic nigrostriatal dopamine signalling via an increase in endogenous GDNF levels is associated with a PFC hypodopaminergic state in *Gdnf* ^*cHyper/cHyper*^;Nestin-Cre mice.

### Mid-gestational increase in endogenous GDNF results in schizophrenia-like behavioural abnormalities

**Fig. 3:**
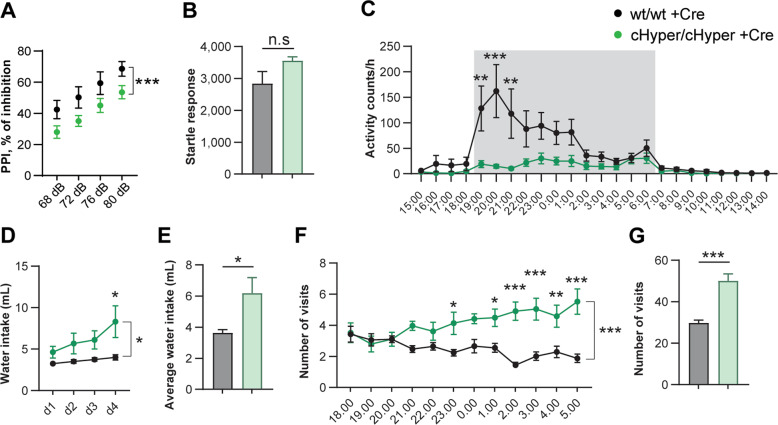
Increased endogenous GDNF results in behavioural abnormalities. See also Supplementary Fig. S3. **A** Pre-pulse inhibition test showing the percentage of inhibition across four different intensities of pre-pulse stimulus. Mean ± SEM. *N* = 6–7 mice per group. Two-way ANOVA. ***p < 0.001 genotype effect. **B** Startle response (arbitrary units) in response to a 120 dB stimulus. Mean ± SEM. *N* = 6–7 mice per group. Welch’s *t*-test. n.s, not significant. **C** Average home cage activity, measured as distance travelled, in individually housed *Gdnf* ^*wt/wt*^;Nestin-Cre and *Gdnf* ^*cHyper/cHyper*^;Nestin-Cre mice over 4 days. Shaded area denotes dark period (lights off), corresponding to the active period in mice. *Gdnf* ^*cHyper/cHyper*^;Nestin-Cre mice lack the initial activity peak observed in wild-type littermate controls. Mean ± SEM. *N* = 13–19 mice per group. Two-way repeated measures ANOVA, Time × Genotype interaction *p* < 0.0001. Sidak’s multiple comparisons test. **p < 0.01; ***p < 0.001. **D** Daily water intake in individually housed *Gdnf* ^*wt/wt*^;Nestin-Cre and *Gdnf* ^*cHyper/cHyper*^;Nestin-Cre mice. Mean ± SEM. *N* = 7–8 mice per group. Two-way repeated measures ANOVA, Genotype effect *p* = 0.0371. Sidak’s multiple comparisons test. *p < 0.05. **E** Average water intake in individually housed *Gdnf* ^*wt/wt*^;Nestin-Cre and *Gdnf* ^*cHyper/cHyper*^;Nestin-Cre mice over 4 days. Mean ± SEM. *N* = 7–8 mice per group. Welch’s *t*-test. *p < 0.05. **F** Number of visits to water bottle compartments during the dark period in individually tracked mice in the IntelliCage setting. Note that unlike *Gdnf* ^*wt/wt*^;Nestin-Cre mice, *Gdnf* ^*cHyper/cHyper*^;Nestin-Cre mice do not exhibit reduced activity towards the end of the active (dark) period. Mean ± SEM. *N* = 9–14 mice per group. Two-way repeated measures ANOVA, Genotype effect *p* < 0.0001. Sidak’s multiple comparisons test. *p < 0.05; **p < 0.01; ***p < 0.001. **G** Total number of visits to water bottle compartments in individually tracked mice in the IntelliCage group-housed setting over 4 days. Mean ± SEM. *N* = 9–14 mice per group. Welch’s *t*-test. ***p < 0.001.

To investigate the behavioural outcome of aberrant dopamine signalling in *Gdnf* ^*cHyper/cHyper*^;Nestin-Cre mice, we used a panel of behavioural tests. We first used the prepulse inhibition (PPI) test, which reflects the ability to successfully integrate and inhibit sensory information. PPI refers to a reduction in startle response in response to an intense stimulus if preceded by a lower intensity stimulus [57]. Individuals with schizophrenia and other schizotypal disorders, as well as animal models with schizophrenia-like features have diminished responses to PPI [58–62], which is believed to predominantly reflect impaired striatal and cortical dopamine transmission [60, 61, 63]. We found that the extent of PPI was significantly reduced in *Gdnf* ^*cHyper/cHyper*^;Nestin-Cre mice compared with controls (Fig. 3A), whereas the startle response was not changed (Fig. 3B), suggesting that altered dopamine signalling was accompanied by a deficit in sensorimotor gating.

Next, we used home-cage monitoring to analyse the pattern of daily activity. Apathy and/or avolition, an inability to initiate and perform routine daily tasks is a core negative symptom of schizophrenia [64–66] and often manifests as prolonged periods of inactivity [67–69]. While not all aspects of avolition can be evaluated in mice, general activity in a familiar environment (a home cage) is relatively easy to assess using the InfraMot system. We found that wild-type animals displayed a well-described activity peak at the onset of the dark period, corresponding to the start of the active period (subjective day) in mice (Fig. 3C). By contrast, *Gdnf* ^*cHyper/cHyper*^;Nestin-Cre mice lacked this initial activity peak and remained less active throughout the day (Fig. 3C). This phenotype was not caused by a general deficit in movement, as evident from normal exploration behaviour in novel environment observed in the open field test (Supplementary Fig. S3A), suggesting that *Gdnf* ^*cHyper/cHyper*^;Nestin-Cre mice exhibit a phenotype resembling apathy or avolition.

Next, based on the observation that 6–20% of individuals with schizophrenia display polydipsia (excessive drinking) [70–72], we measured daily water intake in *Gdnf* ^*cHyper/cHyper*^;Nestin-Cre mice in individually housed male mice and female mice grouped housed in the IntelliCage setting, where the activity of each mouse is tracked with a subcutaneous transponder [73]. We found that compared with controls, *Gdnf* ^*cHyper/cHyper*^;Nestin-Cre mice showed increased water intake in the home cage (Fig. 3D, E) and increased number of visits to water bottle containing corners in IntelliCage (Fig. 3F, G), suggesting a polydipsia-like phenotype. There were no differences between genotypes in tests measuring anxiety (Supplementary Fig. S3B), compulsory behaviour (Supplementary Fig. S3C), learning and memory (Supplementary Fig. S3D, F), motor impulsivity (Supplementary Fig. S3E) or problem solving (Supplementary Fig. S3G).

Together, our results suggest that striatal hyperdopaminergia and PFC hypodopaminergia in *Gdnf* ^*cHyper/cHyper*^;Nestin-Cre mice are associated with specific deficits in sensorimotor gating, daily activity and drinking behaviour, but not with general locomotor activity, anxiety, compulsive behaviour or cognitive function.

### Increase in endogenous GDNF at mid-gestation induces gene expression changes in the PFC

**Fig. 4:**
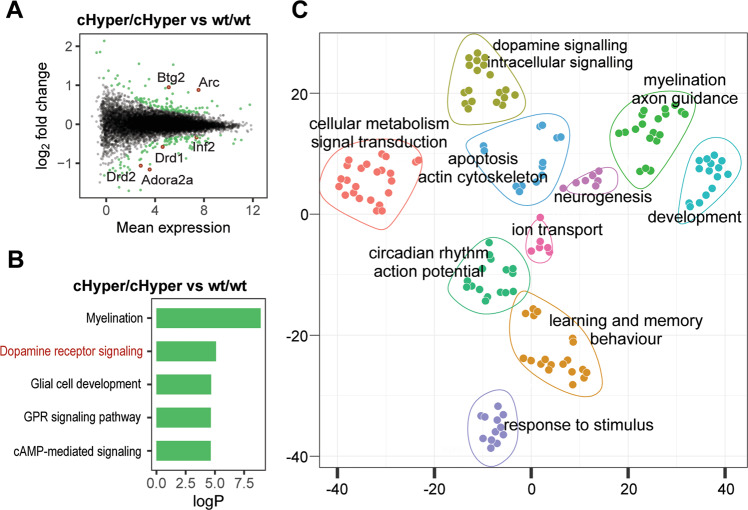
Increased striatal GDNF induces gene expression changes in the PFC. See also Supplementary Figs. S4 and S5. **A** An MA plot showing DE genes (green dots) in the PFC of *Gdnf* ^*cHyper/cHyper*^;Nestin-Cre compared to *Gdnf* ^*wt/wt*^;Nestin-Cre mice. Selected schizophrenia-related DE genes are highlighted in red. **B** Top enriched non-redundant GO biological process categories of DE genes in the PFC of *Gdnf* ^*cHyper/cHyper*^;Nestin-Cre compared to *Gdnf* ^*wt/wt*^;Nestin-Cre mice. **C** Scatter plot of clusters of top 150 enriched GO biological process categories in *Gdnf* ^*cHyper/cHyper*^;Nestin-Cre mice.

To gain a comprehensive understanding of gene expression changes in the PFC, we performed RNAseq analysis of PFC from *Gdnf* ^*cHyper*^;Nestin-Cre mice at 2 months of age. We identified 311 differentially expressed (DE) genes in *Gdnf* ^*wt/cHyper*^;Nestin-Cre mice and 214 genes in *Gdnf* ^*cHyper/cHyper*^;Nestin-Cre mice compared to controls (Supplementary Fig. S4A, B). We found that 58 of the 214 DE genes had previously been associated with schizophrenia in either genome-wide association studies (GWAS) or in transcriptomic analyses from patients (Supplementary Table S1), suggesting that GDNF overexpression recapitulates a subset of the molecular abnormalities underlying schizophrenia. The identified genes included Drd2 and Adora2a, as well as several other genes with established strong association with schizophrenia, including activity regulated cytoskeleton associated protein (Arc), BTG2 anti-proliferation factor 2 (Btg2), and inverted formin, FH2 and WH2 domain containing (Inf2) (Fig. 4A and Supplementary Fig. S4C) [74–87]. Gene Ontology (GO) analysis revealed myelination and dopamine signalling as the most enriched GO terms in the PFC among DE genes in both heterozygous and homozygous mice (Fig. 4B and Supplementary Fig. S4D), in line with gene expression studies on individuals with schizophrenia [7, 58, 75, 88–91]. Similarly, unbiased clustering of enriched GO categories highlighted changes in biological processes related to dopamine system function, myelination, and neuronal development (Fig. 4C and Supplementary Fig. S5). Given that dysregulation of genes belonging to these GO categories is commonly observed in patient studies, our results suggest that the biological processes affected in schizophrenia are also perturbed in *Gdnf* ^*cHyper*^;Nestin-Cre mice.

### Striatal elevation of endogenous GDNF in adults induces cortical hypodopaminergia

**Fig. 5:**
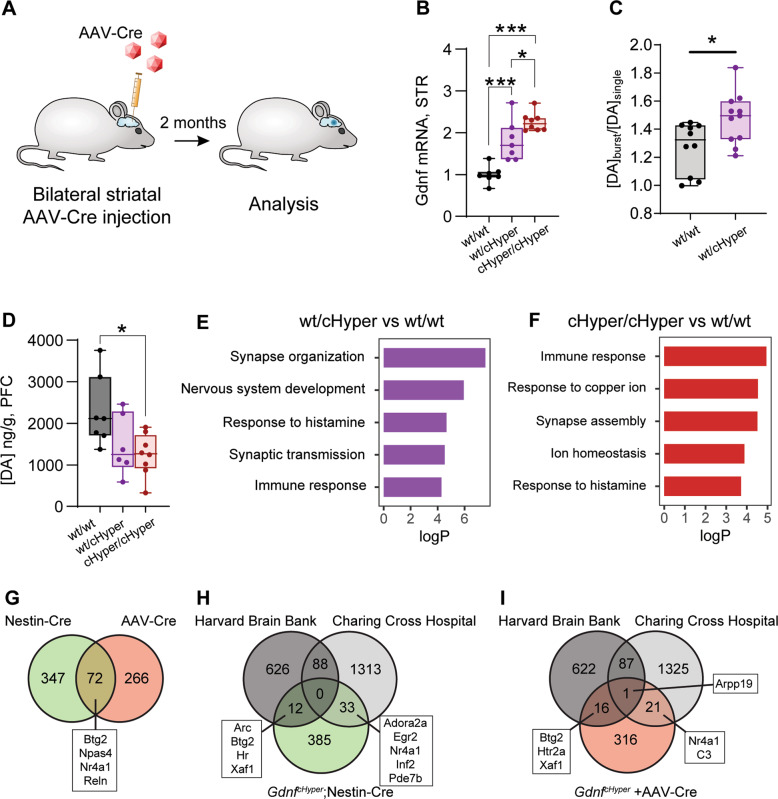
Striatal elevation of endogenous GDNF in adults induces cortical hypodopaminergia. See also Supplementary Fig. S6. **A** Schematic of adult-onset upregulation of endogenous GDNF using AAV-Cre. **B** Striatal Gdnf mRNA levels measured with qPCR. Box plots show median, upper, and lower quartiles, as well as maximum and minimum values. *N* = 7–8 mice per group. One-way ANOVA, Tukey’s multiple comparisons test. *p < 0.05; ***p < 0.001. **C** Striatal dopamine release in response to burst stimulus, relative to single stimulus, measured by fast-scan cyclic voltammetry. Box plots show median, upper and lower quartiles, and maximum and minimum values. *n* = 10–11 striatal slices (from *N* = 6 mice) per group. Unpaired *t*-test. *p < 0.05. **D** Total tissue dopamine levels in the PFC, measured with HPLC. Box plots show median, upper, and lower quartiles, as well as maximum and minimum values. *N* = 6–8 mice per group. One-way ANOVA, Tukey’s multiple comparisons test. *p < 0.05. **E** Top enriched non-redundant GO biological process categories of DE genes in the PFC of *Gdnf* ^*wt/cHyper*^ mice compared to *Gdnf* ^*wt/wt*^ mice. **F** Similar to (**D**) but showing top enriched categories between *Gdnf* ^*cHyper/cHyper*^ and *Gdnf* ^*wt/wt*^ mice. **G** Venn diagram indicating overlapping DE genes in the PFC between *Gdnf* ^*cHyper*^;Nestin-Cre and *Gdnf* ^*cHyper*^ + AAV-Cre mice. Genes differentially expressed in either heterozygous or homozygous mice compared to wild-type controls are shown. **H** Venn diagrams indicating overlapping DE genes in the PFC between post-mortem human PFC RNAseq datasets originating from the Harvard Brain Bank and Charing Cross Hospital, and *Gdnf* ^*cHyper*^;Nestin-Cre mice. **I** Similar to (**G**) but showing the overlap between human datasets and *Gdnf* ^*cHyper*^ + AAV-Cre mice.

Symptoms of schizophrenia typically start in adolescence or adulthood. Furthermore, amphetamine and its derivatives increase nigrostriatal GDNF expression in experimental animals and trigger psychosis in some individuals [21–27]. Thus, we next explored if some of the dopamine system abnormalities found in *Gdnf* ^*cHyper*^;Nestin-Cre mice could be induced by an adult-onset increase of endogenous GDNF expression specifically in the striatum. To that end, we injected AAV-Cre bilaterally into the striata of adult *Gdnf* ^*cHyper*^ mice (Fig. 5A). Two months after striatal AAV-Cre injection, *Gdnf* expression in the striatum was increased in *Gdnf* ^*wt/cHyper*^ and *Gdnf* ^*cHyper/cHyper*^ mice compared with controls (Fig. 5B). FSCV analysis in the striatum of *Gdnf* ^*wt/cHyper*^ mice showed an increase in dopamine release in response to a burst stimulus compared with wild-type littermates (Fig. 5C), and the levels of dopamine and dopamine metabolites in the PFC were significantly reduced, by ~40% (Fig. 5D and Supplementary Fig. S6F, G). There were no significant differences in the total levels of striatal dopamine or dopamine metabolites (Supplementary Fig. S6A–C), or evoked dopamine release and reuptake (Supplementary Fig. S6D, E). Thus, two-fold adult onset increase in endogenous GDNF expression in the striatum is sufficient to trigger schizophrenia-like hypodopaminergia in the PFC, likely via increased activity of striatal dopamine signalling.

To gain insight into molecular response in the PFC, we then analysed PFC gene expression with RNAseq 2 months after adult-onset increase in striatal GDNF. We found 338 DE genes in the PFC of either *Gdnf* ^*wt/cHyper*^ or *Gdnf* ^*cHyper/cHyper*^ mice compared with controls, and identified GO terms related to synaptic transmission and immune response as the predominant affected functional categories, similar to findings from human studies [75, 92, 93] (Fig. 5E, F and Supplementary Fig. S6H–K). Notably, we found that 72 genes were differentially expressed in both adult onset and mid-gestation GDNF elevation models (Fig. 5G), indicating the existence of molecular convergence between the two models.

Next, we compared our PFC RNAseq datasets to two previously published studies describing transcriptional changes in the post-mortem PFC samples from individuals with schizophrenia and matched controls [75, 94]. Of the genes differentially expressed in either *Gdnf* ^*wt/cHyper*^;Nestin-Cre or *Gdnf* ^*cHyper/cHyper*^;Nestin-Cre mice, 45 genes (~10%) overlapped with DE genes in either of these two studies, including Adora2a, Arc, Btg2 and Inf2 (Fig. 5H). Similarly, 38 genes (11%) differentially expressed in the PFC after AAV-Cre-induced adult-onset elevation of striatal GDNF were also identified in at least one of the human studies (Fig. 5I). Therefore, gene expression changes in the PFC of both models share similarities with the findings from human patients.

### A_2A_R antagonism alleviates dopamine system changes in *Gdnf*^*cHyper/cHyper*^;Nestin-Cre mice

**Fig. 6:**
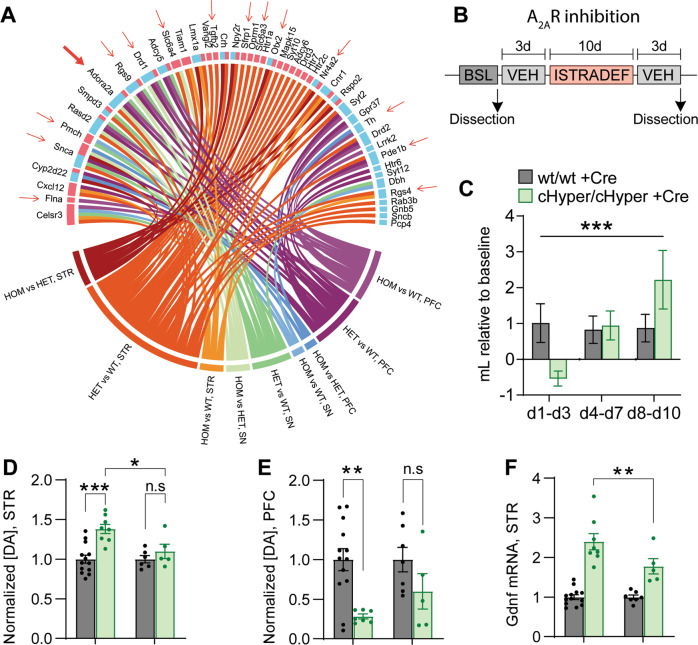
A_2A_R antagonism alleviates dopamine system changes in *Gdnf* ^*cHyper/cHyper*^;Nestin-Cre mice. See also Supplementary Figs. S7 and S8. **A** Chord diagram depicting dopamine system-related genes differentially expressed in the prefrontal cortex (PFC), striatum (STR), or substantia nigra (SN) of *Gdnf* ^*cHyper*^;Nestin-Cre mice. DE genes overlapping with those differentially expressed in *Gdnf* ^*cHyper*^ + AAV-Cre mice are shown with red arrows. Genes downregulated in *Gdnf* ^*cHyper*^ mice compared with wild-type controls are indicated with a blue bar and genes upregulated in *Gdnf* ^*cHyper*^ mice are indicated with a pink bar next to the name of the gene. WT, *Gdnf* ^*wt/wt*^;Nestin-Cre; HET, *Gdnf* ^*wt/cHyper*^;Nestin-Cre; HOM, *Gdnf* ^*cHyper/cHyper*^;Nestin-Cre. **B** Experiment schematic of A_2A_R inhibition using istradefylline. BSL, baseline; VEH, vehicle. See Methods for details. **C** Average daily voluntary consumption of istradefylline-containing solution in the beginning (d1-d3), middle (d4-d7) and end (d8-d10) of the 10-day treatment period. Mean ± SEM. *N* = 5–7 mice per group. Two-way ANOVA Genotype × Period interaction. ***p < 0.001. **D** Striatal dopamine levels normalised to wild-type controls in *Gdnf* ^*wt/wt*^;Nestin-Cre and *Gdnf* ^*cHyper/cHyper*^;Nestin-Cre before and after istradefylline treatment, measured with HPLC. Mean ± SEM. *N* = 5–13 mice per group. One-way ANOVA, Tukey’s multiple comparisons test. *p < 0.05; ***p < 0.001; n.s, not significant. **E** Similar to (**D**) but showing normalised dopamine levels in the prefrontal cortex. Mean ± SEM. *N* = 5–13 mice per group. One-way ANOVA, Tukey’s multiple comparisons test. **p < 0.01; n.s, not significant. **F** Striatal Gdnf mRNA levels in *Gdnf* ^*wt/wt*^;Nestin-Cre and *Gdnf* ^*cHyper/cHyper*^;Nestin-Cre mice after istradefylline treatment. Mean ± SEM. *N* = 5–13 mice per group. One-way ANOVA, Tukey’s multiple comparisons test. **p < 0.01.

To further elucidate the changes caused by increased endogenous GDNF levels that may contribute to phenotypes related to schizophrenia, we performed RNAseq in the striatum and substantia nigra (SN) of *Gdnf* ^*cHyper*^;Nestin-Cre mice at 2–3 months of age and *Gdnf* ^*cHyper*^ mice 2 months after striatal AAV-Cre delivery (Supplementary Fig. S7A, C–J). Due to GDNF’s importance in regulating the midbrain dopamine system, we compared the effects of developmental and adult-onset GDNF upregulation on dopamine system-related gene expression across different brain areas. Between all analysed regions and models, we identified a total of 57 DE dopamine system-related genes (Fig. 6A and Supplementary Fig. S7B). Of these, 15 were differentially expressed after both developmental and adult-onset GDNF elevation, including Adora2a, Drd1, Tyrosine hydroxylase (Th), and Nuclear receptor subfamily 4 group A member 2 (Nr4a2, also known as Nurr1) (Fig. 6A, red arrows, and Supplementary Fig. S7K, L), illustrating a pronounced effect of a 2–3-fold increase in endogenous GDNF on the dopamine system.

Adora2a, encoding for Adenosine A_2A_ receptor (A_2A_R) is a G-protein coupled receptor that regulates the balance of adenosine/dopamine signalling through its interactions with D_2_R [95–97]. Adenosine decreases dopamine signalling through D_2_R, while A_2A_R antagonists, such as caffeine are believed to enhance dopamine signalling through D_2_R [96, 98, 99]. Our RNAseq analysis revealed a downregulation of striatal and PFC Adora2a expression in *Gdnf* ^*cHyper*^*;Nestin-Cre* mice (Figs. 4A, 6A and Supplementary Fig. S7K). This caught our attention, because Adora2a is required for GDNF-stimulated dopamine release in vitro [100]; reduced striatal Adora2a expression has been identified in a subgroup of schizophrenia patients [83]; and some schizophrenia patients voluntarily self-administer excessive amounts of coffee that contains caffeine, a non-selective antagonist for adenosine receptors, as the active ingredient [101, 102]. Finally, a selective A_2A_R antagonist istradefylline [103] has been shown to be effective in an animal model of obsessive-compulsive disorder triggered by a D_2_R agonist [104]. Therefore, we hypothesized that A_2A_R inhibition could alleviate some of the endogenous GDNF-induced schizophrenia-like features.

To address this hypothesis, we tested how istradefylline regulates the dopamine system of *Gdnf* ^*cHyper/cHyper*^;Nestin-Cre mice. First, we analysed voluntary intake of istradefylline-containing solution over a period of 10 days (Fig. 6B). Since *Gdnf* ^*cHyper/cHyper*^;Nestin-Cre mice have higher water intake at baseline (Fig. 3D, G), we measured daily consumption of istradefylline-containing solution and adjusted the concentration of istradefylline for each animal to achieve a consistent dose of 4 mg/kg/day (Supplementary Fig. S8A, B). We found that while *Gdnf* ^*cHyper/cHyper*^;Nestin-Cre mice consumed less istradefylline-containing solution over the first 3 days, they started to self-administer significantly more istradefylline than the control mice towards the end of the 10-day treatment period (Fig. 6C and Supplementary Fig. S8C), consistent with the increased caffeine consumption observed in some individuals with schizophrenia. Next, we analysed the effect of istradefylline on dopamine levels in the striatum and PFC and found that by the end of the treatment period, tissue dopamine levels in these regions were partially normalised (Fig. 6D, E). Reversal of dopamine levels prompted us to investigate the effect of istradefylline on GDNF expression. In cell lines, adenosine signalling increases GDNF expression via multiple cAMP response elements in its promoter [105–108] and, conversely, A_2A_R inhibition downregulates GDNF expression [109]. qPCR analysis revealed that istradefylline downregulated striatal GDNF expression in *Gdnf* ^*cHyper/cHyper*^;Nestin-Cre mice **(**Fig. 6F), suggesting that partial renormalisation of striatal GDNF levels could be triggering the partial normalisation of striatal and, consequently, PFC dopamine levels. Collectively, these results show that selective inhibition of A_2A_R alleviates dopamine system abnormalities both in the PFC and striatum of *Gdnf* ^*cHyper/cHyper*^;Nestin-Cre mice.

### GDNF levels are increased in the CSF of first-episode psychosis patients

**Fig. 7:**
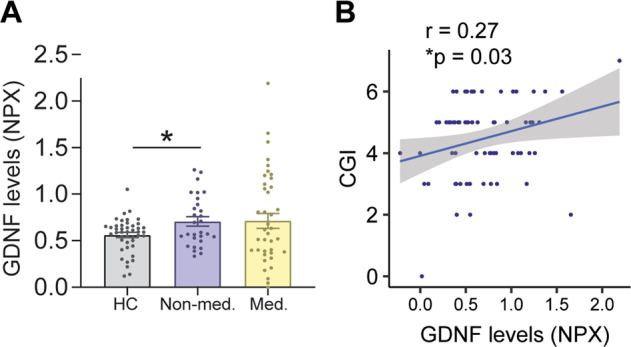
GDNF levels are increased in the CSF of first-episode psychosis patients. See also Supplementary Fig. S9. **A** Comparison of GDNF in cerebrospinal fluid obtained from 44 healthy controls (HCs) and 69 first-episode psychosis (FEP) subjects, including 29 medication-naive individuals (Non-med.) and 40 individuals with prior exposure to antipsychotics (Med.). NPX, Normalized Protein eXpression (see Methods for details). Mean ± SEM. One-way ANOVA, Tukey’s multiple comparisons test. *p < 0.05. **B** Correlation between cerebrospinal fluid GDNF levels and the Clinical Global Impression score in 70 FEP subjects. Pearson product-moment correlation.

To investigate the possible link between GDNF and schizophrenia in humans, we evaluated GDNF levels in the cerebrospinal fluid (CSF) from first episode psychosis (FEP) patients. CSF was collected from 70 FEP patients, who were either fully naive to antipsychotic drugs as per interview and available medical records or had been prescribed an antipsychotic drug for less than 2 months. For comparison, we used CSF from healthy controls (HCs). We found that FEP patients naive to antipsychotic drugs (see Table 1 and Supplementary Table S2 for demographics and clinical characteristics) displayed significantly higher CSF GDNF levels than HCs (Fig. 7A), suggesting that elevated GDNF levels in unmedicated FEP patients are associated with schizophrenia. We also observed an increase in FEP patients with prior history of antipsychotic drugs compared with HCs, but this did not reach statistical significance due to high variance among the patients (Fig. 7A). As a group, FEP patients displayed higher CSF GDNF levels than HCs (Supplementary Fig. S9A), demonstrating that, overall, higher GDNF levels are associated with schizophrenia. We then assessed the relative importance of other potential confounders and found that the single most important factor influencing CSF GDNF levels was indeed case status (Supplementary Fig. S9B). The distribution of GDNF levels was significantly different between healthy controls and FEP subjects, with controls centring more around the mean (Supplementary Fig. S9C).

**Table 1:** Demographics and clinical characteristics of the study participants.

Characteristics	Mean ± SEM		
	Healthy (44)	Patients (70)	*P*-value
Gender (male/female)	25/19	39/31	0.91
BMI	21.75 ± 1.18	23.13 ± 0.59	0.20
Age	26.75 ± 0.89	29.03 ± 0.89	0.26
Nicotine (%)	18%	24%	0.19
DUP (months)	0	9.64 ± 2.04	
*PANSS*			
Positive	–	18.7 ± 0.69	
Negative	–	16.9 ± 0.81	
General	–	37.2 ± 1.25	
Total	–	72.8 ± 2.29	
Levels of functioning			
CGI-S score	–	4.54 ± 0.14	

*P*-values between gender and nicotine difference were calculated with chi-square test. *P*-values between age and BMI were calculated with binomial logistic regression.

*SEM* standard error of mean, *BMI* body mass index, *DUP* duration of untreated psychosis, *PANSS* Positive and Negative Syndrome Scale, *CGI-S* Clinical Global Impression for severity of illness.

To study how GDNF levels associate with the severity of illness in FEP patients, we used the Clinical Global Impression (CGI) for severity of illness (CGI-S) scores. This analysis indicated that CSF GDNF levels correlated with a more severe clinical profile (Fig. 7B, Table 2). No significant differences were observed between FEP patients and HCs regarding age, sex distribution, body mass index, and nicotine usage (Table 1). There were no differences in GDNF levels in the serum of FEP patients compared with controls (Supplementary Fig. S9D).

**Table 2:** Correlations between CSF GDNF and clinical symptoms.

Clinical symptoms	*r*	*P*-value
*PANSS*		
Positive	0.13	0.29
Negative	0.1	0.4
General	0.2	0.09
Total	0.18	0.13
Severity of illness		
CGI-S score	0.27	0.03

*PANSS* Positive and Negative Syndrome Scale, *CGI* Clinical Global Impression.

Subsequently, to evaluate how changes in CSF GDNF levels could reflect GDNF expression in the striatum where GDNF is highly expressed, we utilised raw data from a previously published transcriptomics study analysing striatal gene expression in post-mortem samples from healthy controls and patients with schizophrenia, bipolar disorder, or major depressive disorder [110]. In line with our findings, the results showed a highly significant increase in striatal *GDNF* mRNA levels in post-mortem striatum in individuals with schizophrenia compared to controls (Supplementary Fig. S9E, F). Together, these results show an overall average higher GDNF expression in schizophrenia patients and suggest that elevated GDNF may contribute to the disease, particularly in patients with highest elevation of GDNF levels.

## Discussion

Schizophrenia is a heterogeneous disorder, as indicated by the large number of genetic associations, a wide spectrum of symptoms, and comorbidities with other neuropsychiatric disorders. Recent GWAS analyses suggest that thousands of common genetic variants with small effect sizes contribute to the risk of schizophrenia, and that loci with large effect sizes are very rare [111]. Importantly, by far the most GWAS hits associate with the non-coding genome [112, 113], suggesting that gene regulation, including expression levels play an important role in determining susceptibility to schizophrenia.

GDNF is a neurotrophic factor known for its role in promoting dopamine synthesis and dopamine neuron survival in the midbrain dopamine system [15–18]. Ectopic GDNF overexpression promotes dopamine synthesis in vitro and in vivo [35, 37, 114], and mice with 2–3-fold constitutively elevated endogenous GDNF expression exhibit increased striatal dopamine levels [29]. Although striatal hyperdopaminergia is observed in some schizophrenic and prodromal individuals [8], and the genomic region containing the *GDNF* gene has been associated with schizophrenia by several independent studies [115–117], there have been no studies attempting to investigate the possible contribution of GDNF to dopaminergic abnormalities in schizophrenia.

Here, we report that both mid-gestation- and adult-onset elevation of endogenous GDNF is sufficient to increase presynaptic dopamine function in the striatum and reduce dopamine signalling in the PFC in mice. In FEP patients, GDNF levels are increased in the CSF, and GDNF levels positively correlate with clinical severity scores. It is important to note that the 2–3-fold increase in endogenous GDNF expression achieved in *Gdnf* ^*cHyper*^ mice is considerably higher than the ~20% average increase in GDNF levels in the CSF that we observed in schizophrenia patients. However, in some patients the increase in GDNF is more prominent. Therefore, analysis on how GDNF levels in the CSF relate to various parameters, such as dopamine metabolism, A2AR levels and ligand binding, coffee intake, avolition and polydipsia is an important future perspective.

In mice, we found that increased levels of endogenous GDNF were associated with reduced presynaptic dopamine signalling in the PFC, regardless of whether GDNF was increased during mid-gestation or in the adult. However, the timing of endogenous GDNF upregulation impacted distinct pathways. For example, the predominant affected pathways in the PFC after developmental GDNF elevation included myelination and intracellular signalling, whereas adult-onset increase in endogenous GDNF caused changes in the expression of synaptic genes and immune response (Figs. 4B, C, 5E, F and Supplementary Fig. S4D). Together, these observations suggest that both mid-gestation and adult-onset elevation in endogenous GDNF result in striatal hyperdopaminergia and cortical hypodopaminergia. However, the effect of endogenous GDNF levels on other biological processes is critically dependent on the timing of GDNF expression.

RNAseq analysis identified adenosine 2A receptor as one of the genes differentially expressed in multiple brain regions in *Gdnf* ^*cHyper*^;Nestin-Cre mice. We therefore used istradefylline, a selective first-in-class A_2A_R antagonist to test if reducing A_2A_R activity in *Gdnf* ^*cHyper/cHyper*^;Nestin-Cre mice could improve dopaminergic system abnormalities. We found that *Gdnf* ^*cHyper/cHyper*^;Nestin-Cre mice voluntarily consumed more istradefylline, resembling the increased caffeine intake observed in some individuals with schizophrenia. Ten days of istradefylline administration reduced striatal GDNF expression and partially reversed the developmentally induced striatal hyperdopaminergia and cortical hypodopaminergia in *Gdnf* ^*cHyper/cHyper*^;Nestin-Cre mice. This suggests that selective A_2A_R antagonism may alleviate some of the symptoms of schizophrenia, with a potential specific relevance for a subgroup of patients with high striatal GDNF levels.

**Fig. 8:**
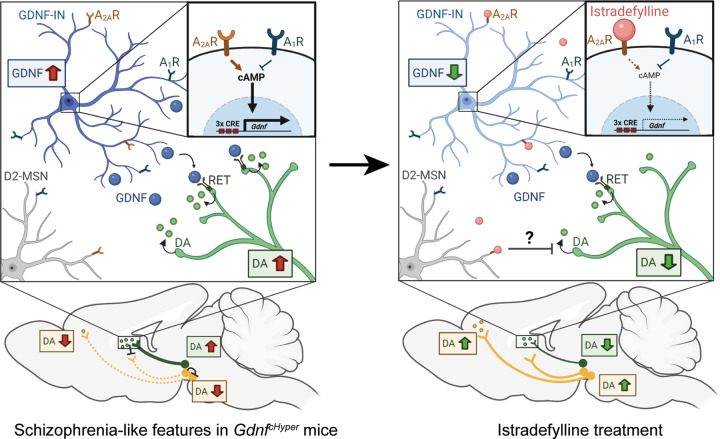
A model showing istradefylline-induced normalization of striatal and PFC dopamine levels in *Gdnf* ^*cHyper/cHyper*^;Nestin-Cre mice by reducing GDNF-induced dopamine release via A_2A_R antagonism. Left: Dopaminergic system abnormalities in *Gdnf* ^*cHyper*^ mice and in individuals with schizophrenia are induced by elevated endogenous GDNF expression in striatal interneurons (blue). In these interneurons, GDNF expression is at least partly regulated by the interplay between A_2A_R-induced and A_1_R-mediated inhibition of cAMP signalling that promotes GDNF expression through cAMP response elements (CRE) in *Gdnf* promoter (inset). Activation of GDNF receptor RET in SN dopamine neurons (green) enhances dopamine release in the dorsal striatum, leading to reduced mesocortical dopamine innervation and dopamine signalling (yellow) in the prefrontal cortex via a yet unknown mechanism. Right: Chronic administration of istradefylline, a specific A_2A_R antagonist, leads to inhibition of A_2A_R in GDNF-expressing interneurons, resulting in reduced intracellular cAMP signalling and reduced *Gdnf* expression (inset). Consequently, striatal GDNF signalling is dampened, leading to reduced dopamine release by SN dopamine neurons and normalization of dopamine signalling in both nigrostriatal and mesocortical dopamine pathways. In addition, A_2A_R antagonism has an acute effect on GDNF-stimulated dopamine release via a currently unknown mechanism. Figure created with BioRender.com.

Why would selective A_2A_R antagonism be advantageous in schizophrenia? In mice, GDNF-producing striatal interneurons express both A_2A_R and A_1_R receptors [118, 119], which have antagonistic functions (Fig. 8). A_2A_R activation triggers cAMP production which has been shown to activate Gdnf transcription in various cell types via cAMP response elements (CRE) in *Gdnf* promoter [106–108, 120]. Given that A_2A_R antagonism acutely reduces GDNF-induced dopamine release in synaptosomes and striatal slices [100], A_2A_R signalling could promote GDNF-triggered dopamine release. Thus, A_2A_R antagonism may alleviate schizophrenia symptoms by two complementary mechanisms: acutely via inhibition of enhanced GDNF-driven striatal dopamine release and chronically via downregulation of striatal GDNF expression (Fig. 8).

One-size-fits-all therapeutic approaches for schizophrenia do not exist. Many patients are refractory to current medication and so have no effective medication, underpinning a need for identifying additional risk factors for a better prediction of treatment response in patient subgroups [121]. In that regard, first episode psychosis patients with high levels of GDNF in the CSF may be of special interest for future research on A_2A_R and RET inhibition therapy. In dopamine neurons, GDNF mainly signals via RET receptor [122, 123], suggesting that RET inhibitors such as Selpercatinib [124] and Pralsetinib [125], currently used in cancer therapy, may carry potential to treat a subgroup of patients with schizophrenia.

It remains to be determined how GDNF levels in the CSF and the brain relate to GDNF levels in the serum, and how changes in GDNF reflect disease status and treatment response. Several studies have shown that treatment with antipsychotics impacts serum GDNF levels [126–130]. While the results are somewhat controversial, some of these reports suggest that antipsychotics increase serum GDNF levels, although it is unknown if GDNF levels are also increased in the brain. Our results suggest that GDNF levels in the serum and CSF may not be directly correlated; thus, the impact of antipsychotics on striatal GDNF expression awaits further investigation. However, in cultured cells, GDNF expression is impacted by signalling through D2 and 5-HT_2A_ receptors that are targeted by second-generation antipsychotics [131–133]. Therefore, it is possible that inhibiting these receptors by antipsychotics directly increases GDNF expression in the brain. However, even if this is the case, any possible downstream changes in dopamine levels would not lead to increased dopamine signalling through D2 receptors, because the latter are blocked by the drug. In that context, increased levels of GDNF could potentially be beneficial in other processes such as adult neurogenesis or neuronal plasticity [134] to support neuronal function in disease. This would be in line with the observation that some atypical antipsychotics increase neurogenesis in adult hippocampus [135], although the underlying mechanisms are unknown. Taken together, the regulation of GDNF levels by antipsychotics and its impact on dopamine signalling and neuronal function in schizophrenia remain important directions for future research.

Lastly, we devised a novel approach to study the effect of an increase in GDNF expression limited to GDNF naturally transcribing cells. Many regulatory genes have a tightly controlled spatiotemporal expression pattern, which is critical to their proper function. Importantly, human studies have shown that as little as a 1.5–2-fold increase in endogenous gene expression levels or biological activity is sufficient to cause neurological disease [136–140]. In addition, while neurotrophic factors, including GDNF, are generally considered to be beneficial in the context of neurological disease, our study demonstrates that the outcomes of neurotrophic factor signalling can be context-dependent and that too high neurotrophic signalling can also drive disease. Therefore, to better understand gene function and therapeutic potential and to create better disease models, it would be advantageous to maintain gene expression from the natural cellular source and at close to physiological expression levels, rather than overexpress with transgenic or viral delivery methods [29, 141] (Supplementary Fig. S10). The levels of more than half of all protein-coding genes are predicted to be negatively regulated through the 3'UTR [142, 143], suggesting that the conditional hypermorph strategy described here is likely to be applicable to other genes. While each approach to study gene function has its limitations, we believe that an ability to conditionally increase gene expression at the post-transcriptional level as implemented in our research, may provide important new opportunities for both basic and translational research.

## Materials and methods

Detailed description of Materials and Methods can be found in Supplementary Methods.

### Experimental model and subject details

#### Human samples

Ethical approval was given by Stockholm Regional Ethics Committee (Dnr 2010/879-31/1). Demographics and clinical characteristics of the study participants are provided in Table 1. Informed consent was obtained from all included subjects.

#### Animals

Animal experiments were conducted according to the 3R principles of the European Union Directive 2010/63/EU and following local laws and regulations. Protocols were authorised by the national Animal Experiment Board of Finland.

### Method details

#### CSF collection and GDNF detection

CSF sampling was performed between 7:45AM and 10:00AM. Samples were centrifuged at 1438 *g* for 10 min at 4 °C. Supernatant was divided into aliquots and stored at −80 °C. The panel ‘Inflammation’ from Olink Bioscience (Uppsala, Sweden) was used to assess protein levels in the CSF.

#### Generation of *Gdnf*^*cHyper*^ knock-in allele

The targeting construct for the *Gdnf* ^*cHyper*^ allele (Supplementary Fig. S1A) contained a 4021 bp 5' homologous arm, a 610 bp cassette containing the bovine growth hormone polyadenylation signal (bGHpA) in an inverted orientation flanked by the FLEx system [46] starting immediately after the stop codon, a 2615 bp Pgk1-puΔtk-bGHpA sequence flanked by Frt sites, and a 2927 bp 3' homologous arm. G4 embryonic stem cells were electroporated with 30 μg of linearized targeting construct. After puromycin selection, colonies were screened by long-range PCR for both 5' and 3' homologous arms and correct PCR products were verified by sequencing. Correctly targeted ES cells were injected into C57BL/N6Crl mouse blastocysts to generate chimeric mice. Germline transmission was achieved by breeding male chimeras with C57BL/N6Crl females. The Pgk1-puΔtk-bGHpA sequence was removed using the CAG-Flp mouse line at F2.

### Quantification and statistical analyses

#### Sample size

Sample size was determined based on preliminary experiments and prior experience with the used assays. No statistical methods were used to predetermine sample size.

#### Rules for stopping data collection

Rules for stopping data collection were not defined.

#### Data inclusion/exclusion criteria and handling of outliers

Data was excluded based on insufficient quality (assessed immediately after acquisition and before analysis).

#### Research subjects or units of investigation

In human studies, research subjects correspond to individual patients or controls. In animal experiments, research subjects correspond to individual animals, except for FSCV experiments, where the experimental units are brain slices.

#### Randomization

Because the genotype of the animal determined the experimental group, randomization to treatment was not possible. To reduce possible bias, only litters including animals from different genotypes were included. In behavioural tests, animals were tested in a random order. Treatments were administered in a random order.

#### Blinding

Experiments were performed by investigators blinded to experimental groups.

#### Statistical analysis

Values are presented as mean ± standard error of the mean. Statistical comparison between two groups was performed using unpaired Student’s *t*-test or Welch’s *t*-test, where appropriate. Multiple comparisons were performed with one-way or two-way analysis of variance (ANOVA), followed by Tukey’s or Sidak’s *post hoc* test. Statistical significance was set at *P* < 0.05.

## Supplementary information

Supplementary Tables

Supplementary Figures

Supplementary Methods

## Data Availability

The GEO accession number for RNA sequencing data is GSE162974.
