# Supplementary material for: Elevated endogenous GDNF induces altered dopamine signalling in mice and correlates with clinical severity in schizophrenia
Source: Mol Psychiatry. 2022 May 26;27(8):3247–61. doi: 10.1038/s41380-022-01554-2 (PMC9708553; doi:10.1038/s41380-022-01554-2)
Supplement: Supplementary file 1 — Supplementary Tables [file 41380_2022_1554_MOESM1_ESM.pdf]

SUPPLEMENTARY TABLES

Elevated endogenous GDNF induces altered dopamine signalling in mice and correlates with clinical severity in schizophrenia

Kärt Mätlik, PhD [1, *], Daniel R. Garton, MSc [1, †], Ana R. Montaño-Rodríguez, MSc [1, †], Soophie Olfat, MSc [1, 2, †], Feride Eren, MSc [3], Laoise Casserly, MSc [1], Anastasios Damdimopoulos, PhD [4], Anne Panhelainen, PhD [5], L. Lauriina Porokuokka, PhD [1], Jaakko J. Kopra, PhD [6], Giorgio Turconi, MSc [1], Nadine Schweizer, PhD [2], Erika Bereczki, PhD [2], Fredrik Piehl, MD, PhD [7], Göran Engberg, PhD [3], Simon Cervenka, MD, PhD [8, 9], T. Petteri Piepponen, PhD [6], Fu-Ping Zhang, MD, PhD [10, 11], Petra Sipilä, PhD [10], Johan Jakobsson, PhD [12], Carl M. Sellgren, MD, PhD [3, 8], Sophie Erhardt, PhD [3], Jaan-Olle Andressoo, PhD [1, 2, *, #]

[1] Department of Pharmacology, Faculty of Medicine, Neuroscience Center & Helsinki Institute of Life Science, University of Helsinki; 00290 Helsinki, Finland.
[2] Division of Neurogeriatrics, Department of Neurobiology, Care Sciences and Society (NVS), Karolinska Institutet; 14183 Huddinge, Sweden.
[3] Department of Physiology and Pharmacology, Karolinska Institutet; 17177 Stockholm, Sweden.
[4] Department of Biosciences and Nutrition, Karolinska Institutet; 14183 Huddinge, Sweden.
[5] Institute of Biotechnology, University of Helsinki; 00014 Helsinki, Finland.
[6] Division of Pharmacology and Pharmacotherapy, Faculty of Pharmacy, University of Helsinki; 00014 Helsinki, Finland.
[7] Department of Clinical Neuroscience, Neuroimmunology Unit, Karolinska Institutet, Karolinska University Hospital; 17177 Stockholm, Sweden.
[8] Centre for Psychiatry Research, Department of Clinical Neuroscience, Karolinska Institutet & Stockholm Health Care Services, Region Stockholm; 17177 Stockholm, Sweden.
[9] Department of Medical Sciences, Psychiatry, Uppsala University; 75185 Uppsala, Sweden.
[10] Research Centre for Integrative Physiology and Pharmacology, Institute of Biomedicine and Turku Center for Disease Modeling, University of Turku; 20520 Turku, Finland.
[11] GM-Unit, Laboratory Animal Center, Helsinki Institute of Life Science, University of Helsinki; 00290 Helsinki, Finland.
[12] Laboratory of Molecular Neurogenetics, Department of Experimental Medical Science, Wallenberg Neuroscience Center and Lund Stem Cell Center, BMC A11, Lund University; 221 84 Lund, Sweden.

Supplementary Table S1. Differentially expressed protein-coding genes in the prefrontal cortex of *Gdnf*$^{cHyper/cHyper}$;Nestin-Cre mice that have been associated with schizophrenia in mouse and/or human studies.

Gene symbol	P-value	Differential expression	Genetic association	Animal model	References
Adora2a	<0.0001	+	+		(1-3)
Adcy7	0,00032		+		(4)
Alas2	0,00048		+		(5)
Aldh1a1	<0.0001	+	+		(3, 6)
Als2cl	<0.0001		+		(7)
Ano2	0,00066		+		(8)
Arc	<0.0001	+	+	+	(3, 9, 10)
Arnt	0,00015		+		(11, 12)
Btg2	<0.0001	+	+		(3, 13)
Cbln4	0,00026	+			(3)
Celsr3	0,00021		+		(4)
Cnp	<0.0001	+			(14)
Col16a1	<0.0001		+		(4)
Col19a1	0,00014		+		(15)
Cpeb1	0,00056		+		(12, 16)
Cyr61	<0.0001	+			(3)
Dennd6b	<0.0001		+		(4)
Dnah6	<0.0001		+		(4)
Drd1	<0.0001	+	+		(11, 17-21)
Drd2	<0.0001	+	+	+	(2, 11, 17, 22-29)
Dusp1	<0.0001	+			(30)
Egr1	0,00029	+	+		(3, 23)
Egr2	<0.0001	+			(3)
Fos	<0.0001		+		(31)
Galnt9	<0.0001	+			(3)
Gpr88	<0.0001		+	+	(32, 33)
Hr	0,00029	+			(3)
Ier2	0,00055		+		(4)
Il18bp	0,00053		+		(34)
Il3ra	<0.0001		+		(35-37)
Inf2	0,00011	+			(3)
Lin28b	<0.0001		+		(16)
Mag	<0.0001	+	+		(38-41)
Masp2	0,00074		+		(4)
Mbp	<0.0001	+	+		(6, 42, 43)
Mobp	<0.0001	+	+		(6, 41, 44, 45)

Mog	0,00047	+	+		(41, 46-49)
Mov10	0,00015		+		(4)
Ndrg1	0,00045	+			(3)
Ndst3	0,00015		+		(50-52)
Npas4	<0.0001	+			(53)
Nr4a1	<0.0001	+			(30, 54)
Pde10a	<0.0001	+	+		(55, 56)
Pde7b	<0.0001	+	+		(3, 57, 58)
Peg3	0,00024		+		(4)
Penk	<0.0001		+		(59, 60)
Pkd1	0,00061	+			(3)
Plekhh1	<0.0001		+		(4)
Plp1	<0.0001	+	+		(41, 61, 62)
Rasd2	<0.0001	+	+	+	(63, 64)
Rtel1	0,00073	+			(3)
Tac1	0,00072	+			(3)
Th	<0.0001		+		(65-67)
Thpo	0,0005	+			(3)
Trf	<0.0001	+	+		(14, 41, 43, 68-70)
Trh	<0.0001		+		(4, 71)
Tspan2	0,00014	+			(3)
Xaf1	<0.0001	+			(3)

Supplementary Table S2, related to Figure 7. Demographics and clinical characteristics of the study participants (healthy controls and drug-naïve first episode psychosis patients).

Supplementary Table S1. Demographics and clinical characteristics of the study participants

Characteristictics	Mean ± s.e.m (n)		P-value
	Healthy Controls (44)	Patients (29)	
Gender (male/female)	25/19	18/11	0.65
BMI	21.75 ± 1.18	23.59 ± 0.67	0.41
Nicotine (%)	18 %	27 %	0.33
DUP (months)	0	12.52 ± 4.26	
Age	26.75 ± 0.89	29.34 ± 1.27	0.15
PANSS			
Positive	–	18.5 ± 0.96	
Negative	–	16.9 ± 1.10	
General	–	36.5 ± 1.99	
Total	–	72 ± 3.36	
Levels of Functioning			
CGI Score	–	4.48 ± 0.21	

P-values between gender and nicotine difference are calculated with chi-square test.
P-values between age and BMI are calculated with binomial logistic regression.

Supplementary Table S3. Oligonucleotide sequences.

Genotyping primers for *Gdnf*^{cHyper} allele	
**F1**	TCTAAGAAAGCATTCCGCTAAACG
**F2**	TTCCAGGGTCAAGGAAGGCAC
**R1**	GGATGCGGTGGGCTCTATG
**R2**	TCCGCCATCTTGGTCCTTATC

qPCR primer sequences	
**Mm Gdnf F**	CGCTGACCAGTGACTCCAATATGC
**Mm Gdnf R**	TGCCGCTTGTTTATCTGGTGACC
**Mm Drd2 F**	ACACACGCTACAGCTCCAAG
**Mm Drd2 R**	GGAGTAGACCACGAAGGCAG
**Mm Gapdh F**	GCCTCGTCCCGTAGACAAAA
**Mm Gapdh R**	ATGAAGGGGTCGTTGATGGC
**Mm Hprt1 F**	CAGTCCCAGCGTCGTGATTA
**Mm Hprt1 R**	TGGCCTCCCATCTCCTTCAT
**Mm Pgk1 F**	TTGGACAAGCTGGACGTGAA
**Mm Pgk1 R**	AACGGACTTGGCTCCATTGT
**Mm Actb F**	CTAAGGCCAACCGTGAAAAG
**Mm Actb R**	ACCAGAGGCATACAGGGACA

REFERENCES

1. Villar-Menendez I, Diaz-Sanchez S, Blanch M, Albasanz JL, Pereira-Veiga T, Monje A, et al. Reduced striatal adenosine A2A receptor levels define a molecular subgroup in schizophrenia. J Psychiatr Res. 2014;51:49-59.
2. Jagannathan K, Calhoun VD, Gelernter J, Stevens MC, Liu J, Bolognani F, et al. Genetic associations of brain structural networks in schizophrenia: a preliminary study. Biol Psychiatry. 2010;68(7):657-66.
3. Maycox PR, Kelly F, Taylor A, Bates S, Reid J, Logendra R, et al. Analysis of gene expression in two large schizophrenia cohorts identifies multiple changes associated with nerve terminal function. Mol Psychiatry. 2009;14(12):1083-94.
4. Fromer M, Pocklington AJ, Kavanagh DH, Williams HJ, Dwyer S, Gormley P, et al. De novo mutations in schizophrenia implicate synaptic networks. Nature. 2014;506(7487):179-84.
5. Dann J, DeLisi LE, Devoto M, Laval S, Nancarrow DJ, Shields G, et al. A linkage study of schizophrenia to markers within Xp11 near the MAOB gene. Psychiatry Res. 1997;70(3):131-43.
6. Ayalew M, Le-Niculescu H, Levey DF, Jain N, Changala B, Patel SD, et al. Convergent functional genomics of schizophrenia: from comprehensive understanding to genetic risk prediction. Mol Psychiatry. 2012;17(9):887-905.
7. Girard SL, Gauthier J, Noreau A, Xiong L, Zhou S, Jouan L, et al. Increased exonic de novo mutation rate in individuals with schizophrenia. Nat Genet. 2011;43(9):860-3.
8. Giacopuzzi E, Gennarelli M, Minelli A, Gardella R, Valsecchi P, Traversa M, et al. Exome sequencing in schizophrenic patients with high levels of homozygosity identifies novel and extremely rare mutations in the GABA/glutamatergic pathways. PLoS One. 2017;12(8):e0182778.
9. Manago F, Mereu M, Mastwal S, Mastrogiacomo R, Scheggia D, Emanuele M, et al. Genetic Disruption of Arc/Arg3.1 in Mice Causes Alterations in Dopamine and Neurobehavioral Phenotypes Related to Schizophrenia. Cell Rep. 2016;16(8):2116-28.
10. Huentelman MJ, Muppana L, Corneveaux JJ, Dinu V, Pruzin JJ, Reiman R, et al. Association of SNPs in EGR3 and ARC with Schizophrenia Supports a Biological Pathway for Schizophrenia Risk. PLoS One. 2015;10(10):e0135076.
11. Ng MY, Levinson DF, Faraone SV, Suarez BK, DeLisi LE, Arinami T, et al. Meta-analysis of 32 genome-wide linkage studies of schizophrenia. Mol Psychiatry. 2009;14(8):774-85.
12. Wu Y, Yao YG, Luo XJ. SZDB: A Database for Schizophrenia Genetic Research. Schizophr Bull. 2017;43(2):459-71.
13. Deng X, Takaki H, Wang L, Kuroki T, Nakahara T, Hashimoto K, et al. Positive association of phencyclidine-responsive genes, PDE4A and PLAT, with schizophrenia. Am J Med Genet B Neuropsychiatr Genet. 2011;156B(7):850-8.
14. Prabakaran S, Swatton JE, Ryan MM, Huffaker SJ, Huang JT, Griffin JL, et al. Mitochondrial dysfunction in schizophrenia: evidence for compromised brain metabolism and oxidative stress. Mol Psychiatry. 2004;9(7):684-97, 43.
15. Liao HM, Chao YL, Huang AL, Cheng MC, Chen YJ, Lee KF, et al. Identification and characterization of three inherited genomic copy number variations associated with familial schizophrenia. Schizophr Res. 2012;139(1-3):229-36.
16. Li Z, Chen J, Yu H, He L, Xu Y, Zhang D, et al. Genome-wide association analysis identifies 30 new susceptibility loci for schizophrenia. Nat Genet. 2017;49(11):1576-83.
17. Allen NC, Bagade S, McQueen MB, Ioannidis JP, Kavvoura FK, Khoury MJ, et al. Systematic meta-analyses and field synopsis of genetic association studies in schizophrenia: the SzGene database. Nat Genet. 2008;40(7):827-34.

18. Abi-Dargham A, Xu X, Thompson JL, Gil R, Kegeles LS, Urban N, et al. Increased prefrontal cortical D(1) receptors in drug naive patients with schizophrenia: a PET study with [(1)(1)C]NNC112. J Psychopharmacol. 2012;26(6):794-805.
19. Pan Y, Yao J, Wang B. Association of dopamine D1 receptor gene polymorphism with schizophrenia: a meta-analysis. Neuropsychiatr Dis Treat. 2014;10:1133-9.
20. Zhu F, Yan CX, Wang Q, Zhu YS, Zhao Y, Huang J, et al. An association study between dopamine D1 receptor gene polymorphisms and the risk of schizophrenia. Brain Res. 2011;1420:106-13.
21. Lee KY, Joo EJ, Ji YI, Kim DH, Park JB, Chung IW, et al. Associations between DRDs and schizophrenia in a Korean population: multi-stage association analyses. Exp Mol Med. 2011;43(1):44-52.
22. Sun J, Kuo PH, Riley BP, Kendler KS, Zhao Z. Candidate genes for schizophrenia: a survey of association studies and gene ranking. Am J Med Genet B Neuropsychiatr Genet. 2008;147B(7):1173-81.
23. Schizophrenia Working Group of the Psychiatric Genomics C. Biological insights from 108 schizophrenia-associated genetic loci. Nature. 2014;511(7510):421-7.
24. Lewis CM, Levinson DF, Wise LH, DeLisi LE, Straub RE, Hovatta I, et al. Genome scan meta-analysis of schizophrenia and bipolar disorder, part II: Schizophrenia. Am J Hum Genet. 2003;73(1):34-48.
25. Pergola G, Di Carlo P, D'Ambrosio E, Gelao B, Fazio L, Papalino M, et al. DRD2 co-expression network and a related polygenic index predict imaging, behavioral and clinical phenotypes linked to schizophrenia. Transl Psychiatry. 2017;7(1):e1006.
26. Vercammen A, Weickert CS, Skilleter AJ, Lenroot R, Schofield PR, Weickert TW. Common polymorphisms in dopamine-related genes combine to produce a 'schizophrenia-like' prefrontal hypoactivity. Transl Psychiatry. 2014;4:e356.
27. Zhan L, Kerr JR, Lafuente MJ, Maclean A, Chibalina MV, Liu B, et al. Altered expression and coregulation of dopamine signalling genes in schizophrenia and bipolar disorder. Neuropathol Appl Neurobiol. 2011;37(2):206-19.
28. Li YC, Kellendonk C, Simpson EH, Kandel ER, Gao WJ. D2 receptor overexpression in the striatum leads to a deficit in inhibitory transmission and dopamine sensitivity in mouse prefrontal cortex. Proc Natl Acad Sci U S A. 2011;108(29):12107-12.
29. Kellendonk C, Simpson EH, Polan HJ, Malleret G, Vronskaya S, Winiger V, et al. Transient and selective overexpression of dopamine D2 receptors in the striatum causes persistent abnormalities in prefrontal cortex functioning. Neuron. 2006;49(4):603-15.
30. Corley SM, Tsai SY, Wilkins MR, Shannon Weickert C. Transcriptomic Analysis Shows Decreased Cortical Expression of NR4A1, NR4A2 and RXRB in Schizophrenia and Provides Evidence for Nuclear Receptor Dysregulation. PLoS One. 2016;11(12):e0166944.
31. Boyajyan A, Zakharyan R, Atshemyan S, Chavushyan A, Mkrtchyan G. Schizophrenia-associated Risk and Protective Variants of c-Fos Encoding Gene. Recent Adv DNA Gene Seq. 2015;9(1):51-7.
32. Del Zompo M, Deleuze JF, Chillotti C, Cousin E, Niehaus D, Ebstein RP, et al. Association study in three different populations between the GPR88 gene and major psychoses. Mol Genet Genomic Med. 2014;2(2):152-9.
33. Logue SF, Grauer SM, Paulsen J, Graf R, Taylor N, Sung MA, et al. The orphan GPCR, GPR88, modulates function of the striatal dopamine system: a possible therapeutic target for psychiatric disorders? Mol Cell Neurosci. 2009;42(4):438-47.

34. Shirts BH, Wood J, Yolken RH, Nimgaonkar VL. Comprehensive evaluation of positional candidates in the IL-18 pathway reveals suggestive associations with schizophrenia and herpes virus seropositivity. Am J Med Genet B Neuropsychiatr Genet. 2008;147(3):343-50.
35. Sun S, Wang F, Wei J, Cao LY, Wu GY, Lu L, et al. Association between interleukin-3 receptor alpha polymorphism and schizophrenia in the Chinese population. Neurosci Lett. 2008;440(1):35-7.
36. Sun S, Wei J, Li H, Jin S, Li P, Ju G, et al. A family-based study of the IL3RA gene on susceptibility to schizophrenia in a Chinese Han population. Brain Res. 2009;1268:13-6.
37. Lencz T, Morgan TV, Athanasiou M, Dain B, Reed CR, Kane JM, et al. Converging evidence for a pseudoautosomal cytokine receptor gene locus in schizophrenia. Mol Psychiatry. 2007;12(6):572-80.
38. Yang YF, Qin W, Shugart YY, He G, Liu XM, Zhou J, et al. Possible association of the MAG locus with schizophrenia in a Chinese Han cohort of family trios. Schizophr Res. 2005;75(1):11-9.
39. Wan C, Yang Y, Feng G, Gu N, Liu H, Zhu S, et al. Polymorphisms of myelin-associated glycoprotein gene are associated with schizophrenia in the Chinese Han population. Neurosci Lett. 2005;388(3):126-31.
40. Felsky D, Voineskos AN, Lerch JP, Nazeri A, Shaikh SA, Rajji TK, et al. Myelin-associated glycoprotein gene and brain morphometry in schizophrenia. Front Psychiatry. 2012;3:40.
41. Tkachev D, Mimmack ML, Ryan MM, Wayland M, Freeman T, Jones PB, et al. Oligodendrocyte dysfunction in schizophrenia and bipolar disorder. Lancet. 2003;362(9386):798-805.
42. Purcell SM, Moran JL, Fromer M, Ruderfer D, Solovieff N, Roussos P, et al. A polygenic burden of rare disruptive mutations in schizophrenia. Nature. 2014;506(7487):185-90.
43. Martins-de-Souza D, Gattaz WF, Schmitt A, Maccarrone G, Hunyadi-Gulyas E, Eberlin MN, et al. Proteomic analysis of dorsolateral prefrontal cortex indicates the involvement of cytoskeleton, oligodendrocyte, energy metabolism and new potential markers in schizophrenia. J Psychiatr Res. 2009;43(11):978-86.
44. Hegyi H. Connecting myelin-related and synaptic dysfunction in schizophrenia with SNP-rich gene expression hubs. Sci Rep. 2017;7:45494.
45. Richetto J, Chesters R, Cattaneo A, Labouesse MA, Gutierrez AMC, Wood TC, et al. Genome-Wide Transcriptional Profiling and Structural Magnetic Resonance Imaging in the Maternal Immune Activation Model of Neurodevelopmental Disorders. Cereb Cortex. 2017;27(6):3397-413.
46. Cannon DM, Walshe M, Dempster E, Collier DA, Marshall N, Bramon E, et al. The association of white matter volume in psychotic disorders with genotypic variation in NRG1, MOG and CNP: a voxel-based analysis in affected individuals and their unaffected relatives. Transl Psychiatry. 2012;2:e167.
47. Barley K, Dracheva S, Byne W. Subcortical oligodendrocyte- and astrocyte-associated gene expression in subjects with schizophrenia, major depression and bipolar disorder. Schizophr Res. 2009;112(1-3):54-64.
48. Sokolov BP. Oligodendroglial abnormalities in schizophrenia, mood disorders and substance abuse. Comorbidity, shared traits, or molecular phenocopies? Int J Neuropsychopharmacol. 2007;10(4):547-55.
49. Liu X, Qin W, He G, Yang Y, Chen Q, Zhou J, et al. A family-based association study of the MOG gene with schizophrenia in the Chinese population. Schizophr Res. 2005;73(2-3):275-80.
50. Xiao X, Luo XJ, Chang H, Liu Z, Li M. Evaluation of European Schizophrenia GWAS Loci in Asian Populations via Comprehensive Meta-Analyses. Mol Neurobiol. 2017;54(6):4071-80.

51. Zhang C, Lu W, Wang Z, Ni J, Zhang J, Tang W, et al. A comprehensive analysis of NDST3 for schizophrenia and bipolar disorder in Han Chinese. Transl Psychiatry. 2016;6:e701.
52. Lencz T, Guha S, Liu C, Rosenfeld J, Mukherjee S, DeRosse P, et al. Genome-wide association study implicates NDST3 in schizophrenia and bipolar disorder. Nat Commun. 2013;4:2739.
53. Alachkar A, Wang L, Yoshimura R, Hamzeh AR, Wang Z, Sanathara N, et al. Prenatal one-carbon metabolism dysregulation programs schizophrenia-like deficits. Mol Psychiatry. 2018;23(2):282-94.
54. Xing G, Zhang L, Russell S, Post R. Reduction of dopamine-related transcription factors Nurr1 and NGFI-B in the prefrontal cortex in schizophrenia and bipolar disorders. Schizophr Res. 2006;84(1):36-56.
55. Tam GW, van de Lagemaat LN, Redon R, Strathdee KE, Croning MD, Malloy MP, et al. Confirmed rare copy number variants implicate novel genes in schizophrenia. Biochem Soc Trans. 2010;38(2):445-51.
56. Boden R, Persson J, Wall A, Lubberink M, Ekselius L, Larsson EM, et al. Striatal phosphodiesterase 10A and medial prefrontal cortical thickness in patients with schizophrenia: a PET and MRI study. Transl Psychiatry. 2017;7(3):e1050.
57. Ikeda M, Tomita Y, Mouri A, Koga M, Okochi T, Yoshimura R, et al. Identification of novel candidate genes for treatment response to risperidone and susceptibility for schizophrenia: integrated analysis among pharmacogenomics, mouse expression, and genetic case-control association approaches. Biol Psychiatry. 2010;67(3):263-9.
58. Ingason A, Giegling I, Cichon S, Hansen T, Rasmussen HB, Nielsen J, et al. A large replication study and meta-analysis in European samples provides further support for association of AHI1 markers with schizophrenia. Hum Mol Genet. 2010;19(7):1379-86.
59. Mikesell MJ, Barron YD, Nimgaonkar VL, Sobell JL, Sommer SS, McMurray CT. Gly(247)-->Asp proenkephalin A mutation is rare in schizophrenia populations. Am J Med Genet. 1997;74(2):213-5.
60. Mikesell MJ, Sobell JL, Sommer SS, McMurray CT. Identification of a missense mutation and several polymorphisms in the proenkephalin A gene of schizophrenic patients. Am J Med Genet. 1996;67(5):459-67.
61. Qin W, Gao J, Xing Q, Yang J, Qian X, Li X, et al. A family-based association study of PLP1 and schizophrenia. Neurosci Lett. 2005;375(3):207-10.
62. English JA, Dicker P, Focking M, Dunn MJ, Cotter DR. 2-D DIGE analysis implicates cytoskeletal abnormalities in psychiatric disease. Proteomics. 2009;9(12):3368-82.
63. Vitucci D, Di Giorgio A, Napolitano F, Pelosi B, Blasi G, Errico F, et al. Rasd2 Modulates Prefronto-Striatal Phenotypes in Humans and 'Schizophrenia-Like Behaviors' in Mice. Neuropsychopharmacology. 2016;41(3):916-27.
64. Liu YL, Fann CS, Liu CM, Chen WJ, Wu JY, Hung SI, et al. RASD2, MYH9, and CACNG2 genes at chromosome 22q12 associated with the subgroup of schizophrenia with non-deficit in sustained attention and executive function. Biol Psychiatry. 2008;64(9):789-96.
65. Srivastava V, Deshpande SN, Thelma BK. Dopaminergic pathway gene polymorphisms and genetic susceptibility to schizophrenia among north Indians. Neuropsychobiology. 2010;61(2):64-70.
66. Kurumaji A, Kuroda T, Yamada K, Yoshikawa T, Toru M. An association of the polymorphic repeat of tetranucleotide (TCAT) in the first intron of the human tyrosine hydroxylase gene with schizophrenia in a Japanese sample. J Neural Transm (Vienna). 2001;108(4):489-95.

67. Meloni R, Laurent C, Campion D, Ben Hadjali B, Thibaut F, Dollfus S, et al. A rare allele of a microsatellite located in the tyrosine hydroxylase gene found in schizophrenic patients. C R Acad Sci III. 1995;318(7):803-9.
68. McCullumsmith RE, Gupta D, Beneyto M, Kreger E, Haroutunian V, Davis KL, et al. Expression of transcripts for myelination-related genes in the anterior cingulate cortex in schizophrenia. Schizophr Res. 2007;90(1-3):15-27.
69. Qu M, Yue W, Tang F, Wang L, Han Y, Zhang D. Polymorphisms of Transferrin gene are associated with schizophrenia in Chinese Han population. J Psychiatr Res. 2008;42(11):877-83.
70. Pennington K, Beasley CL, Dicker P, Fagan A, English J, Pariante CM, et al. Prominent synaptic and metabolic abnormalities revealed by proteomic analysis of the dorsolateral prefrontal cortex in schizophrenia and bipolar disorder. Mol Psychiatry. 2008;13(12):1102-17.
71. Caceda R, Kinkead B, Nemeroff CB. Involvement of neuropeptide systems in schizophrenia: human studies. Int Rev Neurobiol. 2007;78:327-76.
