# Supplementary material for: Elevated endogenous GDNF induces altered dopamine signalling in mice and correlates with clinical severity in schizophrenia
Source: Mol Psychiatry. 2022 May 26;27(8):3247–61. doi: 10.1038/s41380-022-01554-2 (PMC9708553; doi:10.1038/s41380-022-01554-2)
Supplement: Supplementary file 2 — Supplementary Figures [file 41380_2022_1554_MOESM2_ESM.pdf]

SUPPLEMENTARY FIGURES

Elevated endogenous GDNF induces altered dopamine signalling in mice and correlates with clinical severity in schizophrenia

Kärt Mätlik, PhD [1, *], Daniel R. Garton, MSc [1, †], Ana R. Montaño-Rodríguez, MSc [1, †], Soophie Olfat, MSc [1, 2, †], Feride Eren, MSc [3], Laoise Casserly, MSc [1], Anastasios Damdimopoulos, PhD [4], Anne Panhelainen, PhD [5], L. Lauriina Porokuokka, PhD [1], Jaakko J. Kopra, PhD [6], Giorgio Turconi, MSc [1], Nadine Schweizer, PhD [2], Erika Bereczki, PhD [2], Fredrik Piehl, MD, PhD [7], Göran Engberg, PhD [3], Simon Cervenka, MD, PhD [8, 9], T. Petteri Piepponen, PhD [6], Fu-Ping Zhang, MD, PhD [10, 11], Petra Sipilä, PhD [10], Johan Jakobsson, PhD [12], Carl M. Sellgren, MD, PhD [3, 8], Sophie Erhardt, PhD [3], Jaan-Olle Andressoo, PhD [1, 2, *, #]

[1] Department of Pharmacology, Faculty of Medicine, Neuroscience Center & Helsinki Institute of Life Science, University of Helsinki; 00290 Helsinki, Finland.
[2] Division of Neurogeriatrics, Department of Neurobiology, Care Sciences and Society (NVS), Karolinska Institutet; 14183 Huddinge, Sweden.
[3] Department of Physiology and Pharmacology, Karolinska Institutet; 17177 Stockholm, Sweden.
[4] Department of Biosciences and Nutrition, Karolinska Institutet; 14183 Huddinge, Sweden.
[5] Institute of Biotechnology, University of Helsinki; 00014 Helsinki, Finland.
[6] Division of Pharmacology and Pharmacotherapy, Faculty of Pharmacy, University of Helsinki; 00014 Helsinki, Finland.
[7] Department of Clinical Neuroscience, Neuroimmunology Unit, Karolinska Institutet, Karolinska University Hospital; 17177 Stockholm, Sweden.
[8] Centre for Psychiatry Research, Department of Clinical Neuroscience, Karolinska Institutet & Stockholm Health Care Services, Region Stockholm; 17177 Stockholm, Sweden.
[9] Department of Medical Sciences, Psychiatry, Uppsala University; 75185 Uppsala, Sweden.
[10] Research Centre for Integrative Physiology and Pharmacology, Institute of Biomedicine and Turku Center for Disease Modeling, University of Turku; 20520 Turku, Finland.
[11] GM-Unit, Laboratory Animal Center, Helsinki Institute of Life Science, University of Helsinki; 00290 Helsinki, Finland.
[12] Laboratory of Molecular Neurogenetics, Department of Experimental Medical Science, Wallenberg Neuroscience Center and Lund Stem Cell Center, BMC A11, Lund University; 221 84 Lund, Sweden.

Supplementary Figure S1

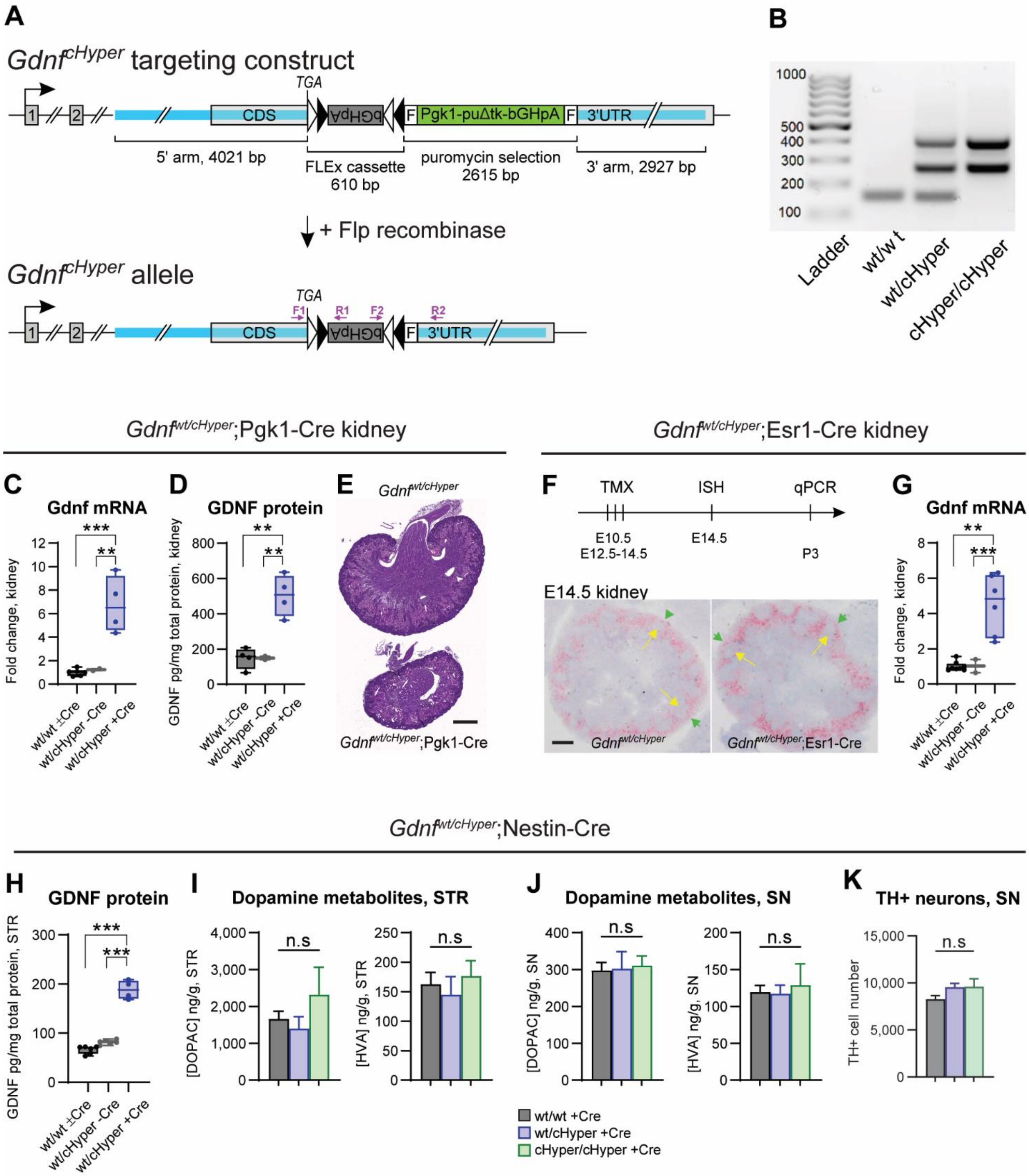

Supplementary Figure S1, related to Figure 1. Generation of *Gdnf* conditional hypermorph (cHyper) allele.

(A) Schematic of the targeting construct (top panel) showing 5' and 3' homologous arms (blue lines), FLEx cassette containing bovine growth hormone polyadenylation signal (bGHpA) in an inverted

orientation (grey box), and puromycin selection marker flanked with Frt sites (F, green box). Crossing to a Deleter Flp mouse line removes the selection cassette and results in $Gdnf^{cHyper}$ allele (bottom panel). Purple arrows indicate the location of genotyping primers. CDS, coding sequence; *TGA*, stop codon.

(B) Representative genotyping image of mice carrying wt/wt, wt/cHyper and cHyper/cHyper alleles.

(C) Gdnf mRNA levels in postnatal day (P3) kidneys of $Gdnf^{cHyper}$;Pgk1-Cre mice, measured with qPCR. Box plots show median, upper and lower quartiles, and maximum and minimum values. N=2-6 per group. One-way ANOVA, Tukey's multiple comparisons test. **$p < 0.01$; ***$p < 0.001$.

(D) GDNF protein levels in P3 kidneys of $Gdnf^{cHyper}$;Pgk1-Cre mice. Box plots show median, upper and lower quartiles, and maximum and minimum values. N=2-4 per group. One-way ANOVA, Tukey's multiple comparisons test. **$p < 0.01$.

(E) A representative image of kidney hematoxylin-eosin staining at P3, demonstrating a severe reduction in kidney size in $Gdnf^{wt/cHyper}$;Pgk1-Cre mice. Scale bar 500 µm.

(F) Timeline of tamoxifen (TMX) treatment (top panel) and a representative *in situ* hybridization (ISH) image (bottom panel) from the kidney at embryonic day 14.5 (E14.5) using RNAscope probe against Gdnf mRNA, demonstrating normal Gdnf expression pattern in $Gdnf^{wt/cHyper}$;Esr1-Cre mice. Green arrows indicate GDNF-expressing metanephric mesenchyme and yellow arrows indicate ureteric buds. Scale bar 100 µm.

(G) Gdnf mRNA expression in P3 kidneys after tamoxifen injection. Box plots show median, upper and lower quartiles, and maximum and minimum values. N=2-8 per group. One-way ANOVA, Tukey's multiple comparisons test. **$p < 0.01$; ***$p < 0.001$.

(H) GDNF protein levels in the striatum of adult $Gdnf^{cHyper}$;Nestin-Cre mice, measured with ELISA and normalised to total protein content. Box plots show median, upper and lower quartiles, and maximum and minimum values. N=4-6 per group. One-way ANOVA, Tukey's multiple comparisons test. ***$p < 0.001$.

(I) Total levels of DOPAC (left) and HVA (right) in the striatum, measured with HPLC. Mean ± SEM. N=4-10 mice per group. One-way ANOVA, Tukey's multiple comparisons test. n.s, not significant.

(J) Total levels of DOPAC (left) and HVA (right) in the substantia nigra, measured with HPLC. Mean ± SEM. N=5-10 mice per group. One-way ANOVA, Tukey's multiple comparisons test. n.s, not significant.

(K) Stereological quantification of the number of tyrosine hydroxylase (TH)-positive neurons in the substantia nigra. Mean ± SEM. N=8-11 mice per group. One-way ANOVA, Tukey's multiple comparisons test. n.s, not significant.

Supplementary Figure S2

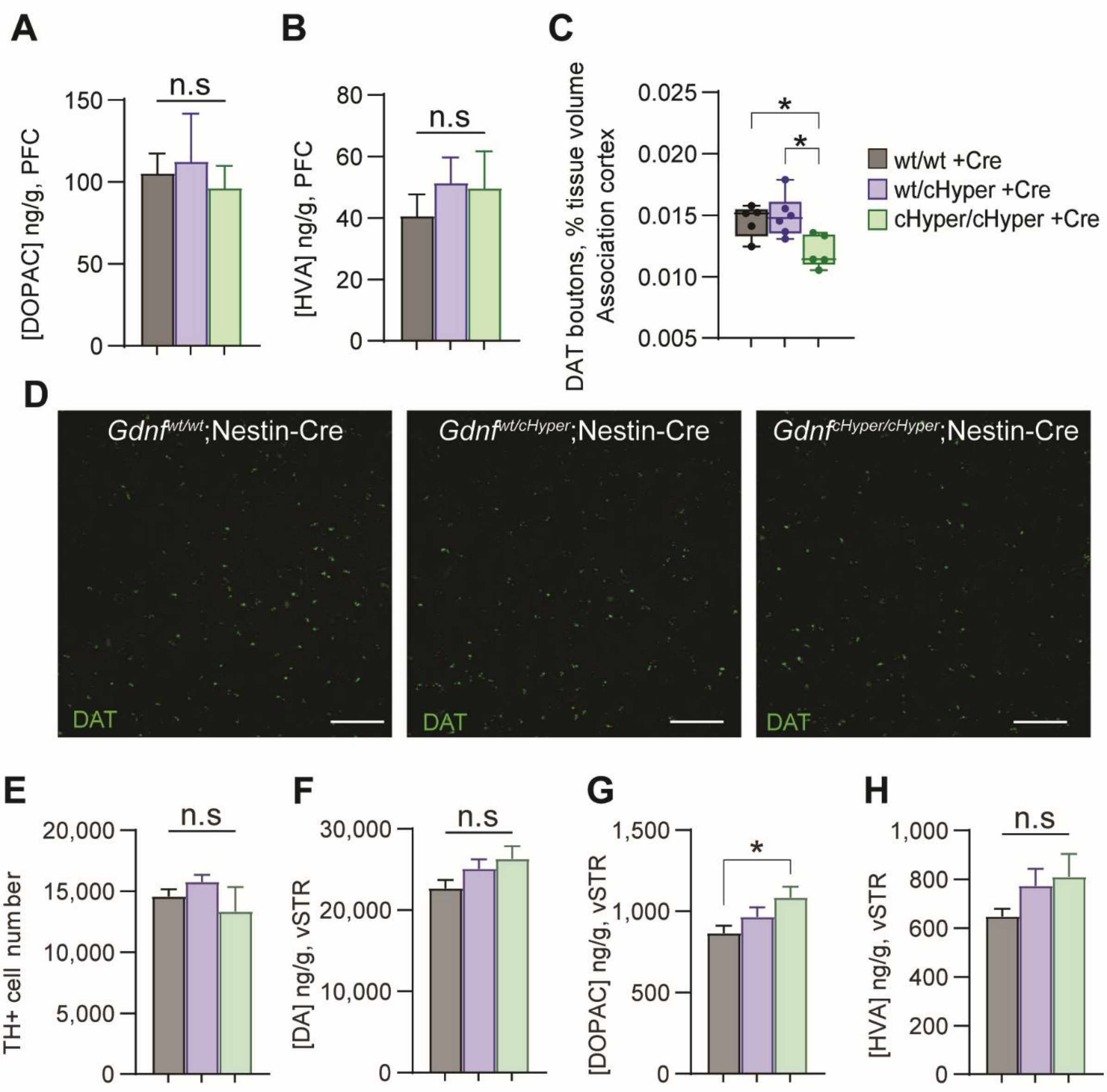

Supplementary Figure S2, related to Figure 2. Increased endogenous GDNF levels reduce dopamine signalling in the PFC.

(A) Total levels of DOPAC in the prefrontal cortex, measured with HPLC. Mean ± SEM. N=5-10 mice per group. One-way ANOVA, Tukey's multiple comparisons test. n.s, not significant.

(B) Total levels of HVA in the prefrontal cortex, measured with HPLC. Mean ± SEM. N=5-10 mice per group. One-way ANOVA, Tukey's multiple comparisons test. n.s, not significant.

(C) Quantification of the number of dopamine transporter (DAT)-positive synaptic boutons in the association cortex in the PFC. Box plots show median, upper, and lower quartiles, as well as maximum and minimum values. N=4-6 mice per group (average values from n=2-3 slices per animal). One-way ANOVA, Tukey's multiple comparisons test. *$p < 0.05$.

(D) Representative images of immunohistochemistry for DAT showing dopamine synapses in the association cortex in the PFC. Scale bar 50 µm.

(E) Stereological quantification of the number of tyrosine hydroxylase (TH)-positive neurons in the ventral tegmental area (VTA). Mean ± SEM. N=3-6 mice per group. One-way ANOVA, Tukey's multiple comparisons test. n.s, not significant.

(F) Total levels of dopamine in the ventral striatum, measured with HPLC. Mean ± SEM. N=8-13 mice per group. One-way ANOVA, Tukey's multiple comparisons test. n.s, not significant.

(G) Total levels of DOPAC in the ventral striatum, measured with HPLC. Mean ± SEM. N=8-13 mice per group. One-way ANOVA, Tukey's multiple comparisons test. *$p < 0.05$.

(H) Total levels of HVA in the ventral striatum, measured with HPLC. Mean ± SEM. N=8-13 mice per group. One-way ANOVA, Tukey's multiple comparisons test. n.s, not significant.

Supplementary Figure S3

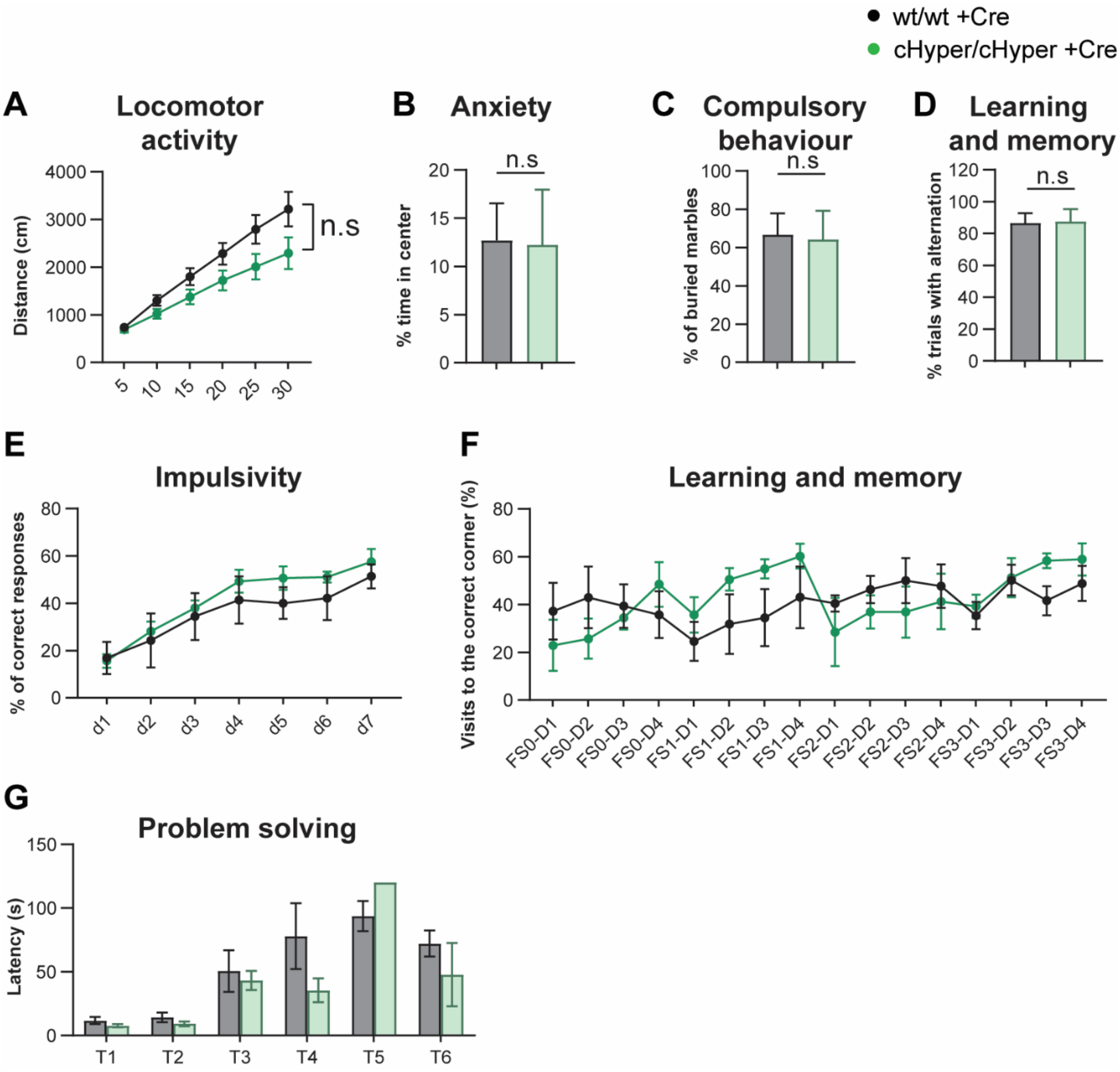

Supplementary Figure S3, related to Figure 3. Increased endogenous GDNF results in behavioural abnormalities.

(A) Cumulative locomotor activity (distance travelled, cm) in the open field test, shown in 5-min blocks over 30 min. Mean ± SEM. N=12-19 mice per group. Two-way repeated measures ANOVA.

(B) Percentage of time spent in the center of the open field arena, indicative of anxiety-like behaviour. Mean ± SEM. N=12-13 mice per group. *t*-test. n.s., not significant.

(C) Percentage of buried marbles in the marble burying test, indicative of compulsory behaviour. Mean ± SEM. N=6-7 mice per group. Mann-Whitney test. n.s, not significant.

(D) Percentage of trials with alternation in the T-maze test. Mean ± SEM. N=4-5 mice per group. Mann-Whitney test. n.s, not significant.

(E) Percent of correct responses in the IntelliCage motor impulsivity task, where mice were trained to wait a delay period of variable length before attempting to access a water bottle. Mean ± SEM. N=5-6 mice per group. Two-way repeated measures ANOVA.

(F) Percent of correct corner visits in the IntelliCage flexible sequencing task evaluating learning and memory. Mean ± SEM. N=4 mice per group. Two-way repeated measures ANOVA.

(G) Latency to exit across 6 trials (T1-T6) in the puzzle box text. Mean ± SEM. N=4-5 mice per group. Two-way ANOVA. Genotype effect p=0.2214 (n.s).

Supplementary Figure S4

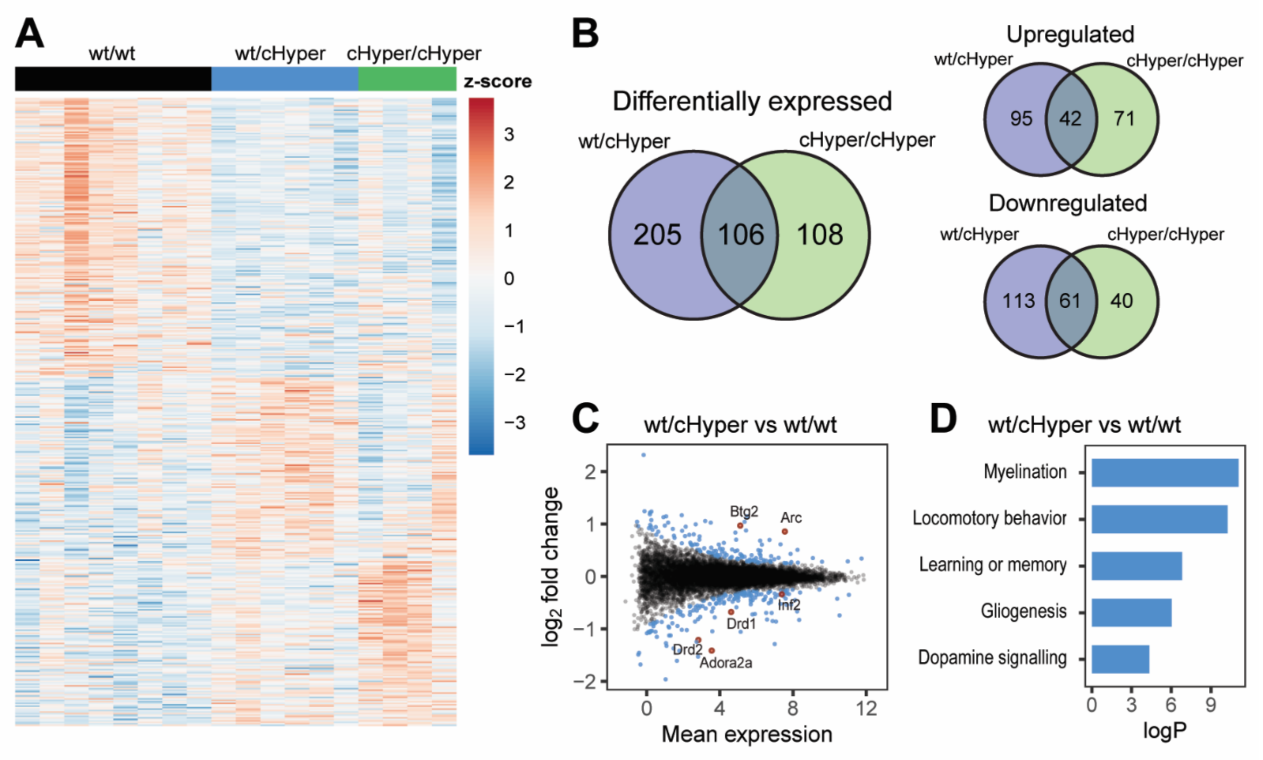

Supplementary Figure S4, related to Figure 4. Increased endogenous GDNF in *Gdnf*^{cHyper};Nestin-Cre mice induces gene expression changes in the PFC.

(A) Heatmap of DE genes in an RNAseq experiment from the PFC of *Gdnf*^{cHyper};Nestin-Cre mice, showing variation between the samples. Differentially expressed (DE) genes between any comparison are included.

(B) Venn diagrams showing the number of all DE genes, and significantly up- and downregulated genes in *Gdnf*$^{wt/cHyper}$;Nestin-Cre (wt/cHyper) and *Gdnf*$^{cHyper/cHyper}$;Nestin-Cre (cHyper/cHyper) mice compared to *Gdnf*$^{wt/wt}$;Nestin-Cre mice.

(C) An MA plot showing DE genes (blue dots) in the PFC of *Gdnf*$^{wt/cHyper}$;Nestin-Cre compared to *Gdnf*$^{wt/wt}$;Nestin-Cre mice. Selected schizophrenia-related DE genes are highlighted in red.

(D) Top enriched non-redundant GO biological process categories of DE genes in the PFC of *Gdnf*$^{wt/cHyper}$;Nestin-Cre compared to *Gdnf*$^{wt/wt}$;Nestin-Cre mice.

Supplementary Figure S5

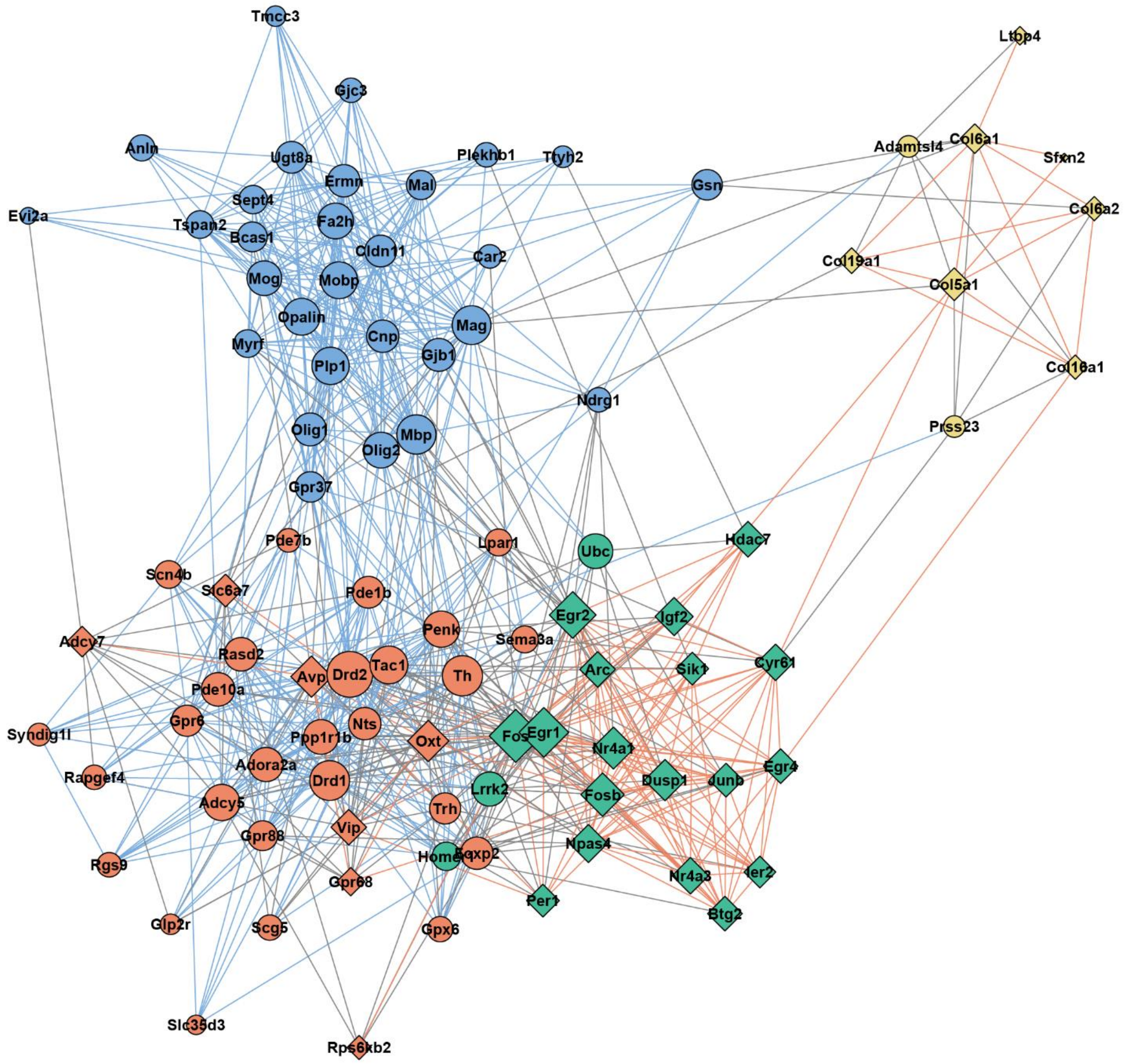

Supplementary Figure S5, related to Figure 4. Gene expression interactome in mice with increased endogenous GDNF.

Interactome of genes found significant in the PFC of *Gdnf*$^{cHyper/cHyper}$;Nestin-Cre compared to *Gdnf*$^{wt/wt}$;Nestin-Cre mice. Genes in 4 most highly enriched clusters are shown. Colours of the nodes (genes) denote different clusters. Genes upregulated in *Gdnf*$^{cHyper/cHyper}$;Nestin-Cre PFC are marked with a diamond shape and downregulated genes are marked with a round shape. Links between nodes are coloured according to the direction of the regulation: links between two upregulated genes are shown in red, links between downregulated genes are shown in blue, and links between genes with different direction of regulation are shown in grey. The size of the nodes indicates the number of connections.

Supplementary Figure S6

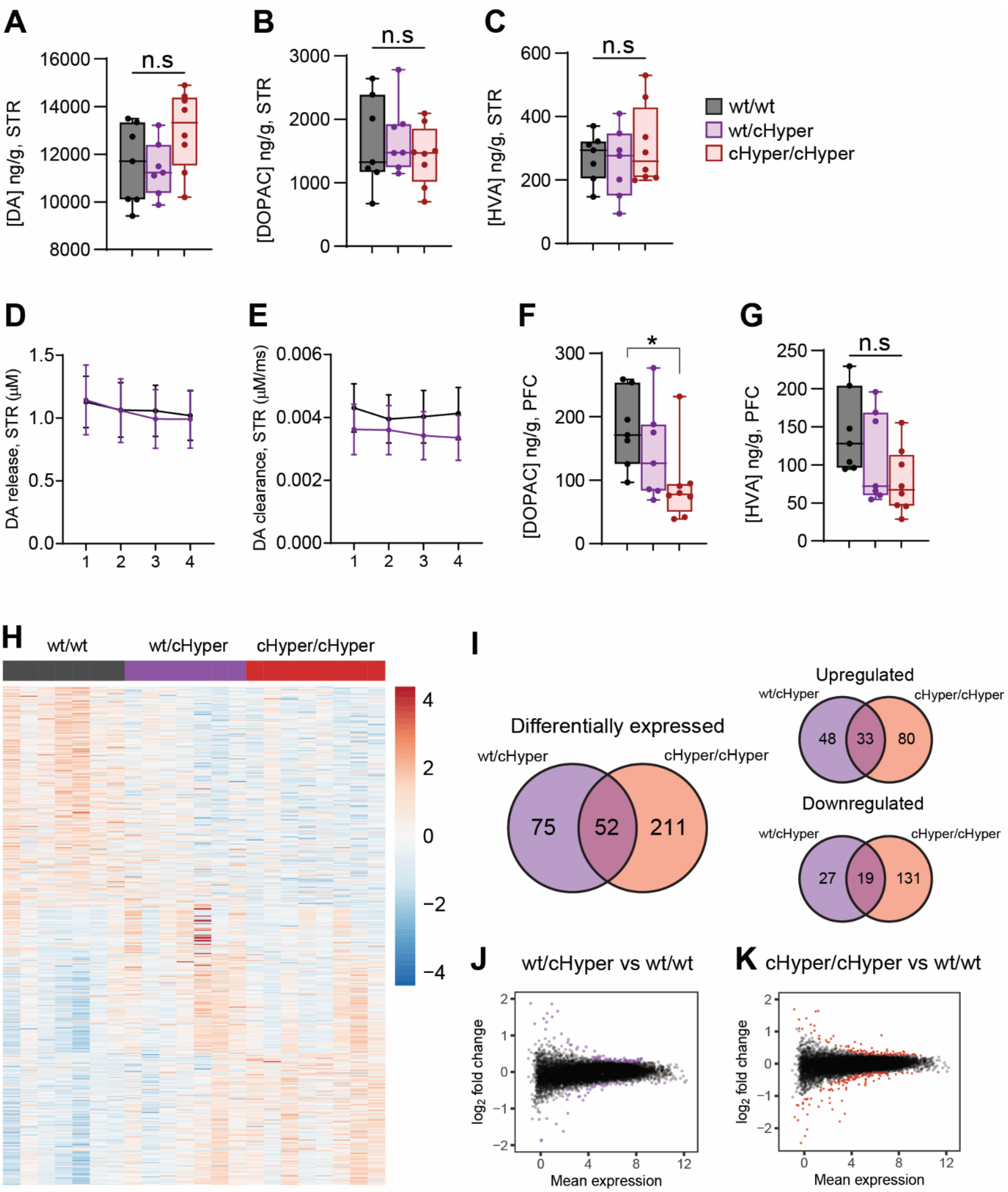

Supplementary Figure S6, related to Figure 5. Striatal elevation of endogenous GDNF in adults induces cortical hypodopaminergia.

(A) Total dopamine levels in the striatum, measured with HPLC. Box plots show median, upper, and lower quartiles, as well as maximum and minimum values. N=7-8 mice per group. One-way ANOVA, Tukey's multiple comparisons test. n.s., not significant.

(B) Total DOPAC levels in the striatum, measured with HPLC. Box plots show median, upper, and lower quartiles, as well as maximum and minimum values. N=7-8 mice per group. One-way ANOVA, Tukey's multiple comparisons test. n.s., not significant.

(C) Total HVA levels in the striatum, measured with HPLC. Box plots show median, upper, and lower quartiles, as well as maximum and minimum values. N=7-8 mice per group. One-way ANOVA, Tukey's multiple comparisons test. n.s., not significant.

(D) Stimulated dopamine release in striatal slices across four consecutive stimulations, measured by fast-scan cyclic voltammetry. Mean ± SEM. n=10-11 striatal slices (from N=6 mice) per group. Two-way repeated measures ANOVA, Sidak's multiple comparisons test.

(E) Similar to (D) but showing the kinetics of dopamine re-uptake. Mean ± SEM. n=10-11 striatal slices (from N=6 mice) per group. Two-way repeated measures ANOVA, Sidak's multiple comparisons test.

(F) Total DOPAC levels in the PFC, measured with HPLC. Box plots show median, upper, and lower quartiles, as well as maximum and minimum values. N=7-8 mice per group. One-way ANOVA, Tukey's multiple comparisons test. *$p < 0.05$.

(G) Total HVA levels in the PFC, measured with HPLC. Box plots show median, upper, and lower quartiles, as well as maximum and minimum values. N=7-8 mice per group. One-way ANOVA, Tukey's multiple comparisons test. n.s., not significant.

(H) Heatmap of DE genes in RNAseq experiment from the PFC of $Gdnf^{cHyper}$ +AAV-Cre mice, showing variation between the samples. Genes differentially expressed (DE) in any comparison were included.

(I) Venn diagrams showing the number of all DE genes, and significantly up- and downregulated genes in $Gdnf^{wt/cHyper}$ +AAV-Cre (wt/cHyper) and $Gdnf^{cHyper/cHyper}$ +AAV-Cre (cHyper/cHyper) mice compared with $Gdnf^{wt/wt}$ +AAV-Cre mice.

(J) An MA plot showing DE genes (purple dots) in $Gdnf^{wt/cHyper}$ +AAV-Cre compared to $Gdnf^{wt/wt}$ +AAV-Cre mice.

(K) An MA plot showing DE genes (red dots) in $Gdnf^{cHyper/cHyper}$ +AAV-Cre compared to $Gdnf^{wt/wt}$ +AAV-Cre mice.

Supplementary Figure S7

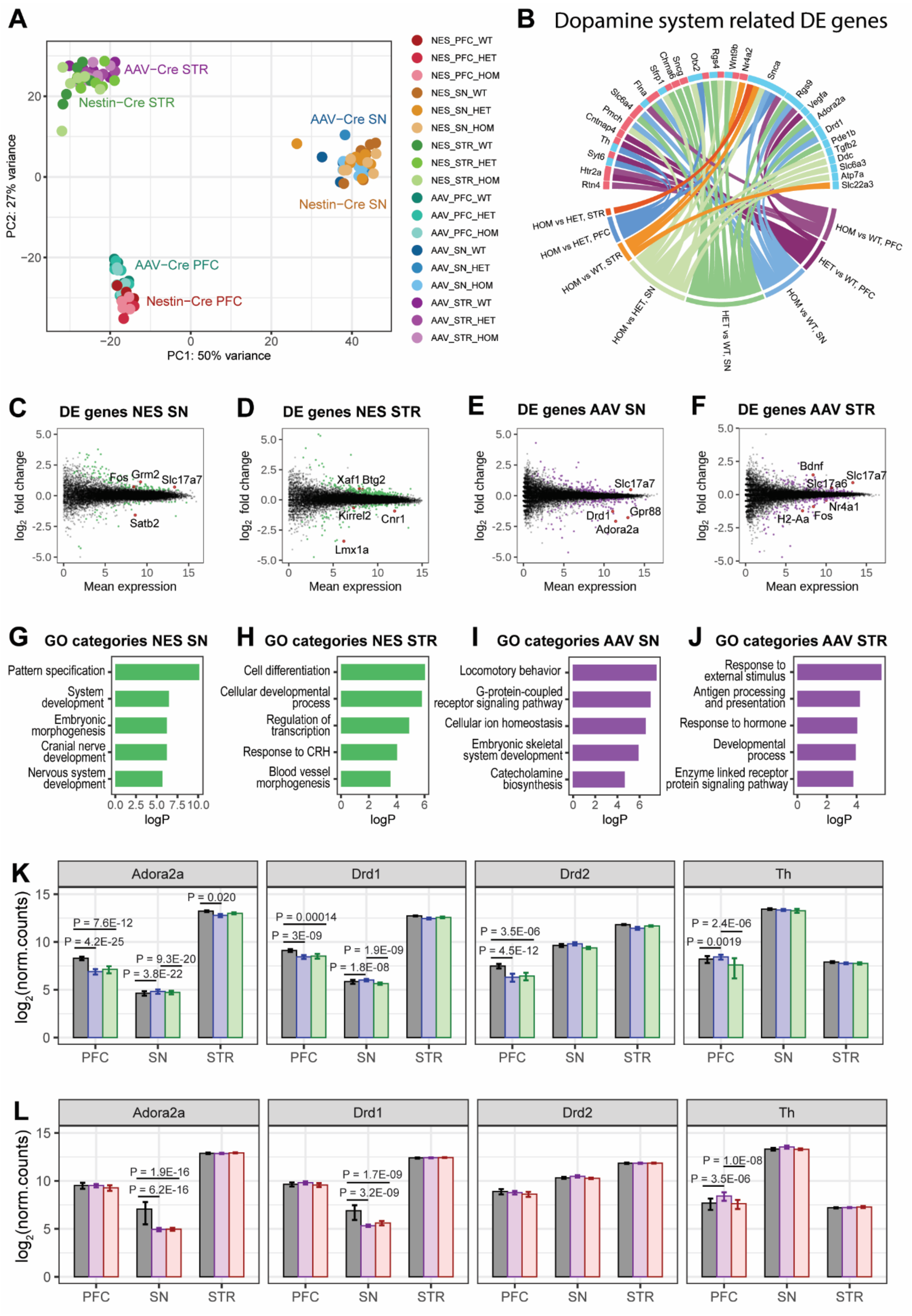

Supplementary Figure S7, related to Figure 6.

(A) Principal component analysis of analysed brain regions: prefrontal cortex (PFC), substantia nigra (SN) and striatum (STR) in *Gdnf*^{cHyper} +AAV-Cre (AAV) and *Gdnf*^{cHyper};Nestin-Cre (NES) mice. WT, wild-type (wt/wt); HET, heterozygous (wt/cHyper); HOM, homozygous (cHyper/cHyper).

(B) A chord diagram depicting dopamine system related genes differentially expressed in the PFC, STR or SN of *Gdnf*^{cHyper} +AAV-Cre mice. Genes downregulated in *Gdnf*^{cHyper} mice compared with wild-type controls are indicated with a blue bar and genes upregulated in *Gdnf*^{cHyper} mice are indicated with a pink bar next to the name of the gene.

(C) An MA plot showing DE genes (green dots) in the SN of *Gdnf*$^{cHyper/cHyper}$;Nestin-Cre compared to *Gdnf*$^{wt/wt}$;Nestin-Cre mice. Selected DE genes are highlighted with red circles.

(D) An MA plot showing DE genes (green dots) in the STR of *Gdnf*$^{cHyper/cHyper}$;Nestin-Cre compared to *Gdnf*$^{wt/wt}$;Nestin-Cre mice. Selected DE genes are highlighted with red circles.

(E) An MA plot showing DE genes (purple dots) in the SN of *Gdnf*$^{cHyper/cHyper}$ +AAV-Cre compared to *Gdnf*$^{wt/wt}$ +AAV-Cre mice. Selected DE genes are highlighted with red circles.

(F) An MA plot showing DE genes (purple dots) in the STR of *Gdnf*$^{cHyper/cHyper}$ +AAV-Cre compared to *Gdnf*$^{wt/wt}$ +AAV-Cre mice. Selected DE genes are highlighted with red circles.

(G) Top enriched non-redundant GO biological process categories in *Gdnf*^{cHyper};Nestin-Cre SN. Genes differentially expressed between any genotypes were included.

(**H**) Top enriched non-redundant GO biological process categories in *Gdnf*^{cHyper};Nestin-Cre STR. Genes differentially expressed between any genotypes were included.

(I) Top enriched non-redundant GO biological process categories in *Gdnf*^{cHyper} +AAV-Cre SN. Genes differentially expressed between any genotypes were included.

(J) Top enriched non-redundant GO biological process categories in *Gdnf*^{cHyper} +AAV-Cre STR. Genes differentially expressed between any genotypes were included.

(K) RNA expression levels of Adora2a, Drd1, Drd2 and Th in *Gdnf*^{cHyper};Nestin-Cre mice based on RNAseq. Mean ± SEM. N=4-8. Adjusted p-values are obtained from edgeR differential expression analysis.

(L) RNA expression levels of Adora2a, Drd1, Drd2 and Th in *Gdnf*^{cHyper} +AAV-Cre mice based on RNAseq. Mean ± SEM. N=7-8. Adjusted p-values are obtained from edgeR differential expression analysis.

Supplementary Figure S8

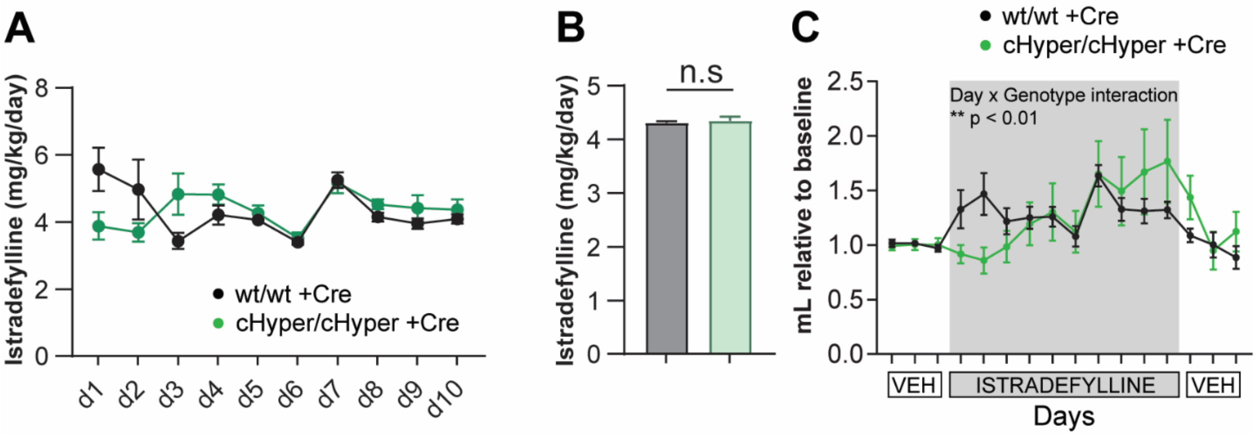

Supplementary Figure S8, related to Figure 6. $A_{2A}R$ antagonism alleviates dopamine system changes in *Gdnf*$^{cHyper/cHyper}$;Nestin-Cre mice.

(A) Istradefylline dose (mg/kg/day) consumed each day in both groups during the 10-day treatment period. Mean ± SEM. N=5-7 mice per group. Two-way repeated measures ANOVA.

(B) Average istradefylline dose (mg/kg/day) consumed in each group over the 10-day treatment period. Mean ± SEM. N=5-7 mice per group. Welch's *t*-test. n.s, not significant.

(C) Voluntary daily intake of istradefylline-containing solution during 10-day treatment period. VEH, vehicle. Mean ± SEM. N=5-7 mice per group. Two-way repeated measures ANOVA, Sidak's multiple comparisons test. Genotype × Day interaction $^{**}p < 0.01$.

Supplementary Figure S9

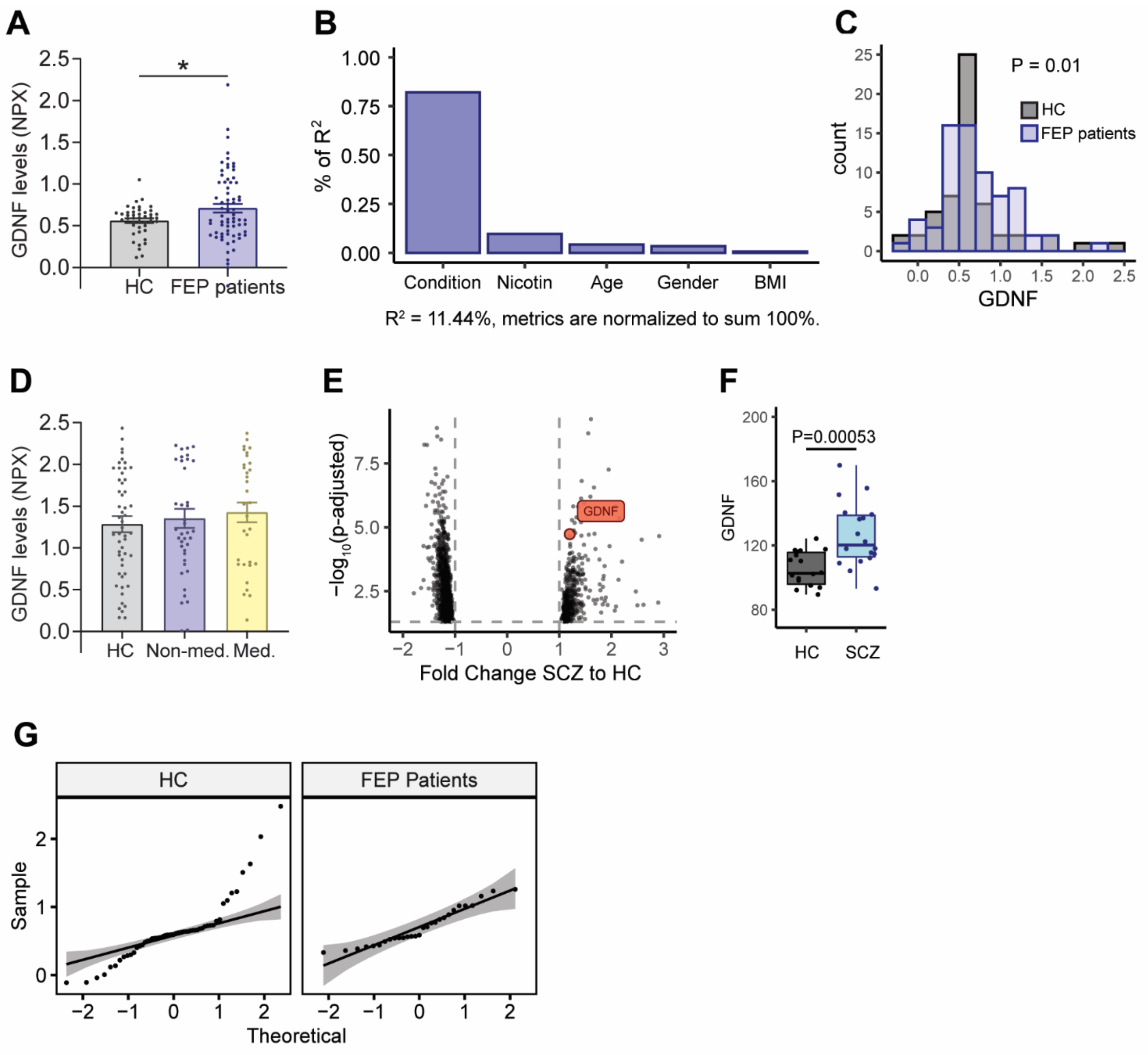

Supplementary Figure S9, related to Figure 7. GDNF levels are increased in the CSF of first-episode psychosis patients.

(A) Comparison of GDNF in cerebrospinal fluid obtained from 44 healthy controls (HCs) and 70 first-episode psychosis (FEP) subjects. NPX, Normalized Protein eXpression (see Methods for details). Mean ± SEM. Welch's t-test. *$p < 0.05$.

(B) R package "relaimpo" output assessing relative importance of potential confounders. Relative importance is assigned by averaging over orders of condition, nicotine, age, gender and BMI.

(C) Distribution of GDNF levels in HCs and FEP patients.

(D) Comparison of GDNF in serum obtained from 44 healthy controls (HCs) and 69 first-episode psychosis (FEP) subjects, including 29 medication-naïve individuals (Non-med.) and 40 individuals with prior exposure to antipsychotics (Med.). NPX, Normalized Protein eXpression (see Methods for details). Mean ± SEM. One-way ANOVA.

(E) Volcano plot showing differentially expressed genes in post-mortem striatal samples from patients with schizophrenia (SCZ) relative to healthy controls (HC; data from Lanz *et al.*, 2019).

(F) *GDNF* mRNA levels in patients with schizophrenia (SCZ) compared with healthy controls (HC; data from Lanz *et al.*, 2019).

(G) Quantile-Quantile Plot to identify outliers in HCs and FEP patients.

Supplementary Figure S10

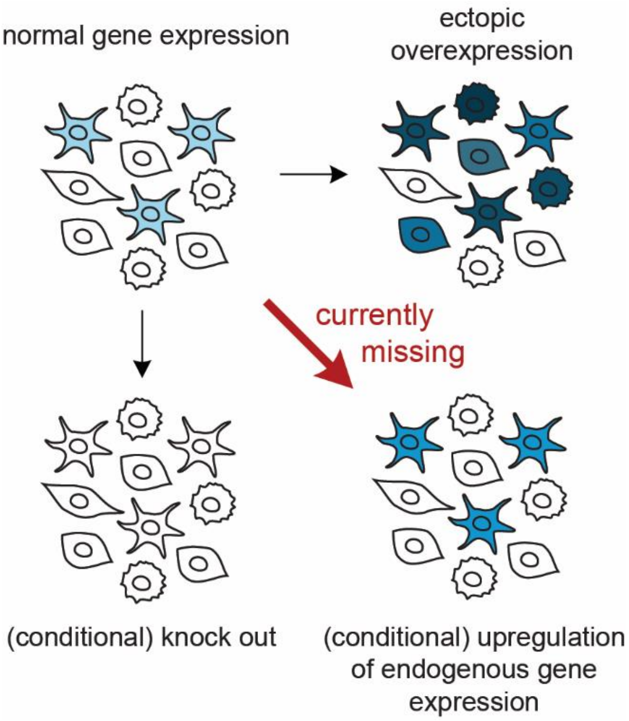

Supplementary Figure S10 Schematic of genetic targeting approaches for studying gene function. Knock-out and conditional knock-out strategies are used to delete or inhibit normal gene expression. Studying the outcome of enhanced gene function is either achieved with agonizing drugs (available for a few hundred human proteins), or by ectopic overexpression using transgenic animals or viral delivery. The ability to increase endogenous gene expression limited to naturally expressing cells is currently not available.

Supplementary Figure S11

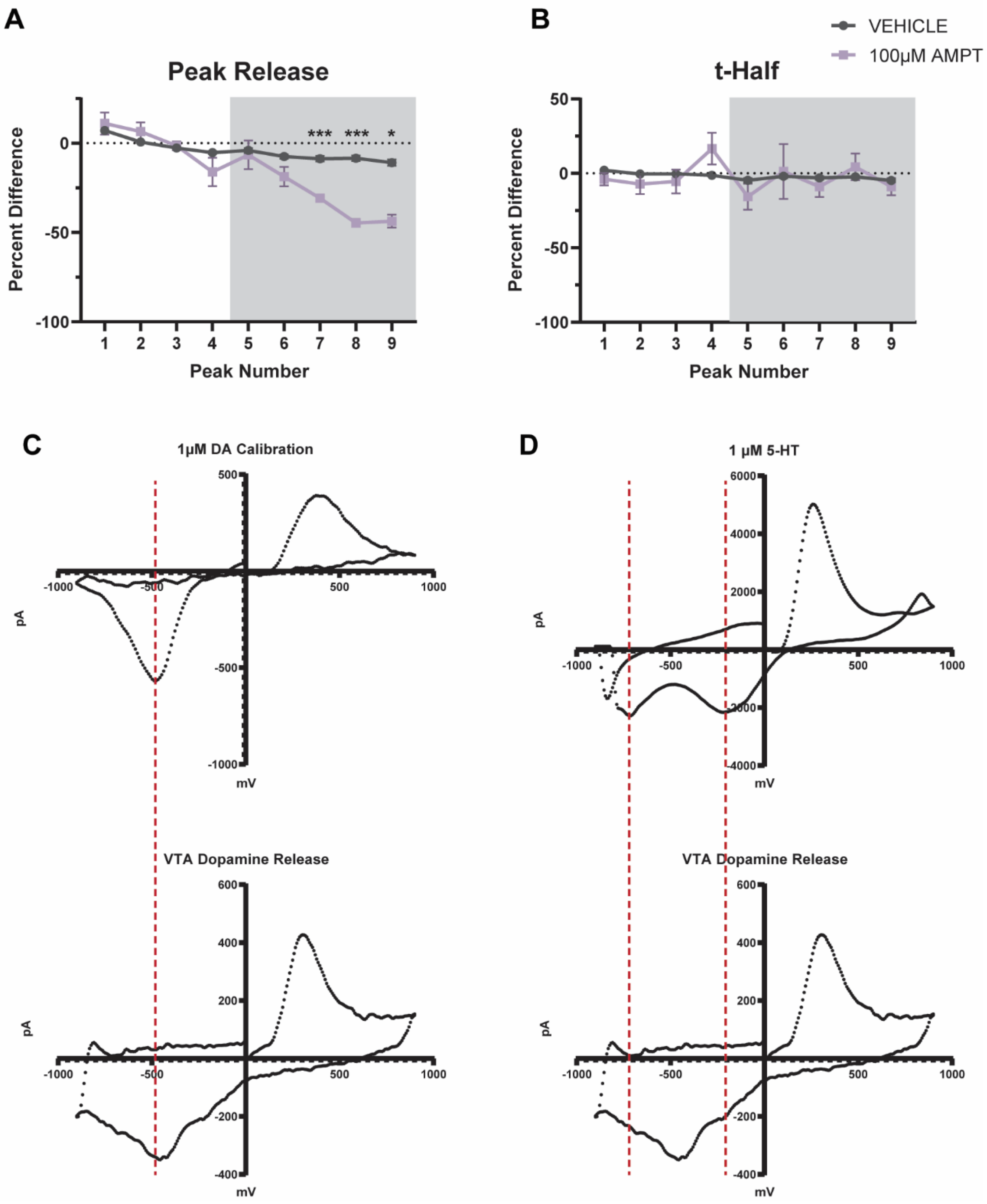

Supplementary Figure S11 Validation of fast-scan cyclic voltammetry for quantifying dopamine release and reuptake.

(A) Quantification of dopamine release in striatal slices treated with vehicle (N=17) or tyrosine hydroxylase inhibitor AMPT (N=3). Mean ± SEM. Between each electrically evoked peak is a two-minute recovery time. Two-way repeated measures ANOVA, Holm-Sidak's multiple comparisons test. *$p<0.05$; ***$p<0.001$. Shaded area indicates bath perfusion of 100 µM AMPT in ACSF or ACSF alone (Vehicle).

(B) Dopamine reuptake kinetics in striatal slices after treatment with vehicle (N=17) or AMPT (N=3). Mean ± SEM. Between each electrically stimulated peak is a two-minute recovery time. Shaded area indicates bath perfusion of 100 μM AMPT in ACSF or ACSF alone (Vehicle).

(C) Upper panel: Background-subtracted FSCV voltammogram with 1 μM dopamine. Lower panel: An example voltammogram from the VTA. Dashed line indicates reduction peak typical for dopamine around -450 mV, present in the VTA voltammogram.

(D) Upper panel: Background-subtracted FSCV voltammogram with 1 μM 5-HT. Lower panel: An example voltammogram from the VTA. Dashed lines indicate dual reduction peaks typical for serotonin around -200 mV and -750 mV, which are notably absent from the VTA voltammogram.
