# Supplementary material for: Elevated endogenous GDNF induces altered dopamine signalling in mice and correlates with clinical severity in schizophrenia
Source: Mol Psychiatry. 2022 May 26;27(8):3247–61. doi: 10.1038/s41380-022-01554-2 (PMC9708553; doi:10.1038/s41380-022-01554-2)
Supplement: Supplementary file 3 — Supplementary Methods [file 41380_2022_1554_MOESM3_ESM.pdf]

SUPPLEMENTARY MATERIALS AND METHODS

Elevated endogenous GDNF induces altered dopamine signalling in mice and correlates with clinical severity in schizophrenia

Kärt Mätlik, PhD [1,*], Daniel R. Garton, MSc [1,†], Ana R. Montaño-Rodríguez, MSc [1,†], Soophie Olfat, MSc [1,2,†], Feride Eren, MSc [3], Laoise Casserly, MSc [1], Anastasios Damdimopoulos, PhD [4], Anne Panhelainen, PhD [5], L. Lauriina Porokuokka, PhD [1], Jaakko J. Kopra, PhD [6], Giorgio Turconi, MSc [1], Nadine Schweizer, PhD [2], Erika Bereczki, PhD [2], Fredrik Piehl, MD, PhD [7], Göran Engberg, PhD [3], Simon Cervenka, MD, PhD [8,9], T. Petteri Piepponen, PhD [6], Fu-Ping Zhang, MD, PhD [10,11], Petra Sipilä, PhD [10], Johan Jakobsson, PhD [12], Carl M. Sellgren, MD, PhD [3,8], Sophie Erhardt, PhD [3], Jaan-Olle Andressoo, PhD [1,2,*,#]

[1] Department of Pharmacology, Faculty of Medicine, Neuroscience Center & Helsinki Institute of Life Science, University of Helsinki; 00290 Helsinki, Finland.
[2] Division of Neurogeriatrics, Department of Neurobiology, Care Sciences and Society (NVS), Karolinska Institutet; 14183 Huddinge, Sweden.
[3] Department of Physiology and Pharmacology, Karolinska Institutet; 17177 Stockholm, Sweden.
[4] Department of Biosciences and Nutrition, Karolinska Institutet; 14183 Huddinge, Sweden.
[5] Institute of Biotechnology, University of Helsinki; 00014 Helsinki, Finland.
[6] Division of Pharmacology and Pharmacotherapy, Faculty of Pharmacy, University of Helsinki; 00014 Helsinki, Finland.
[7] Department of Clinical Neuroscience, Neuroimmunology Unit, Karolinska Institutet, Karolinska University Hospital; 17177 Stockholm, Sweden.
[8] Centre for Psychiatry Research, Department of Clinical Neuroscience, Karolinska Institutet & Stockholm Health Care Services, Region Stockholm; 17177 Stockholm, Sweden.
[9] Department of Medical Sciences, Psychiatry, Uppsala University; 75185 Uppsala, Sweden.
[10] Research Centre for Integrative Physiology and Pharmacology, Institute of Biomedicine and Turku Center for Disease Modeling, University of Turku; 20520 Turku, Finland.
[11] GM-Unit, Laboratory Animal Center, Helsinki Institute of Life Science, University of Helsinki; 00290 Helsinki, Finland.
[12] Laboratory of Molecular Neurogenetics, Department of Experimental Medical Science, Wallenberg Neuroscience Center and Lund Stem Cell Center, BMC A11, Lund University; 221 84 Lund, Sweden.

Resource Availability

Data and Code Availability. The GEO database accession number for the RNA sequencing data reported in this paper is GSE162974. Other datasets and code used in the present study are available from the corresponding author on reasonable request.

Experimental Model and Subject Details

Human samples. Ethical approval was given by Stockholm Regional Ethics Committee (Dnr 2010/879-31/1). Demographics and clinical characteristics of the study participants are provided in **Table 1**. Informed consent was obtained from all included subjects. As part of the Karolinska Schizophrenia Project (KaSP) longitudinal study, FEP patients (defined as patients with a first treatment contact due to psychotic symptoms) were recruited from four psychiatric clinics located in Stockholm, Sweden: Psykiatri Nordväst, Norra Stockholms Psykiatri, Södra Stockholms Psykiatri, and PRIMA Vuxenpsykiatri. Upon recruitment, all patients in KaSP underwent an assessment by at least two-board certified psychiatrists and the Structured Clinical Interview for DSM-IV Axis I Disorders. Further assessments of the clinical characterization were done using PANSS, the Global Assessment of Functioning (GAF) scale, CGI, the Alcohol Use Disorders Identification Test (AUDIT), and the Drug Use Disorders Identification Test (DUDIT). Subjects with signs of neurological disease, or other severe somatic illness, were excluded. This decision was taken based on a brain magnetic resonance imaging (MRI) examination, the clinical interview (complemented with a medical charge review as appropriate), a physical examination, and a blood laboratory screening. Based on the clinical interview, AUDIT, DUDIT, and urine laboratory screening subjects with ongoing or previous drug addiction or history of drug abuse were also excluded. In the current study, a total of 70 FEP patients were included, with these subjects being recruited between March 2011 and March 2019. Patients included in the study were classified as 1) fully naïve to antipsychotics as per interview or available medical history, or 2) exposed to antipsychotic treatment for less than 2 months. At recruitment, the following DSM-IV diagnoses were established: schizophrenia (n= 26), paranoid schizophrenia (n=14), disorganized schizophrenia (n=2), delusional disorders (n=3), acute and transient psychotic disorder (n=2), psychosis not-otherwise-specified (n= 20), schizoaffective syndrome (n= 1), major depressive disorder (single episode, severe with

psychotic features) (n=1) and no diagnosis (n= 1). As part of the study protocol, a re-assessment was also performed after approximately 18 months and the following DSM-IV diagnoses were established: schizophrenia (n= 21), paranoid schizophrenia (n=17), disorganized schizophrenia (n=2), delusional disorders (n=4), acute and transient psychotic disorder (n=1), psychosis not-otherwise-specified (n= 4), schizoaffective syndrome (n= 3), major-depressive disorder (n=1) and no diagnosis (n= 4). Thirteen patients did not participate in the follow-up examination.

The recruitment of HCs was done by advertisement. All HCs underwent a physical examination, blood, and urine laboratory screening, and a brain MRI examination. Exclusion of current or previous psychiatric illness was done by using The Mini International Neuropsychiatric Interview (MINI), and a first-degree relative with a psychotic illness, as well as the previous or current use of illegal drugs, were defined as exclusion criteria.

Animals. All animal experiments were conducted according to the 3R principles of the European Union Directive 2010/63/EU governing the care and use of experimental animals and following local laws and regulations (Finnish Act on the Protection of Animals Used for Scientific or Educational Purposes (497/2013), Government Decree on the Protection of Animals Used for Scientific or Educational Purposes (564/2013)). The protocols were authorised by the national Animal Experiment Board of Finland (license numbers ESAVI/11198/04.10.07/2014 and ESAVI/12046/04.10.07/2017). B6.Cg-Tg(Nes-cre)1Kln/J mice (referred to as Nestin-Cre) were obtained from the Jackson Laboratory (RRID:IMSR_JAX:003771). B6.C-Tg(Pgk1-cre)1Lni/CrsJ mice (61), referred to as Pgk1-Cre, were obtained from the Jackson Laboratory (RRID: IMSR_JAX:020811). Tg(CAG-cre/Esr1*)5Amc/J mice (62), referred to as Esr1-Cre, were obtained from the Jackson Laboratory (RRID: IMSR_JAX:004453). Mice were maintained in a 129Ola/ICR/C57bl6 mixed genetic background. The mice were group-housed in a specific pathogen-free stage with *ad libitum* access to food and water under a 12-h light-dark cycle (lights on at 6 a.m.) at a relative humidity of 50-60 % and at a room temperature of 21±1 °C. Bedding (aspen chips, Tapvei) and nest material (Tapvei) were changed weekly and wooden blocks (Tapvei) were provided for enrichment. All behavioural tests, tissue collections and processing were performed by researchers who were blind to the genotypes of the animals, and in all

experiments, wild-type littermates were used as controls. Both males and females were used (details provided below). ARRIVE guidelines are followed for reporting animal research.

Method Details

CSF collection. CSF sampling was performed between 7:45AM and 10:00AM after a night of rest. To drip 18 ml CSF into a plastic test tube (protected from light), a disposable atraumatic needle (22G Sprotte, Geisingen, Germany) was inserted at the L 4-5 level with all individuals stationed in the right decubitus position. All samples were centrifuged at 1438*g* for 10 minutes at 4 °C (Sigma 5810R, Eppendorf, Hamburg, Germany at 3500 r.p.m.) to separate cells and supernatant. The obtained supernatant was then divided into 10 aliquots that were frozen at −80 °C.

Detection of GDNF in human CSF. The inflammation protein biomarker panel 'Inflammation' from Olink Bioscience (Uppsala, Sweden) was used to assess protein levels in CSF obtained from recruited subjects in the KaSP as part of a larger study. GDNF protein is one of the protein markers in this panel described in more detail at https://www.olink.com/products/inflammation/. In brief, the assay is based on multiplex proximity extension assay technology (PEA), and 92 protein markers can be measured by microtiter plates in the PEA assay. In each well there are DNA-labeled antibody probes of 96 pairs. PEA assay uses pairs of oligonucleotide-linked antibodies with an affinity for each other. Binding of the target proteins and antibody brings PEA probes in close proximity and it leads to the start of DNA polymerization. The new DNA sequence produced is the marker for the targeted protein and is obtained by real-time PCR and then quantified. After normalizing the PCR results for inter- and intra-run variation by using internal control and interplate control, processed data was delivered in an arbitrary unit of Olink called Normalized Protein eXpression (NPX). NPX is in a log2-scale and represents a relative quantification between samples.

To determine if HC samples and FEP patients have a similar distribution we performed a Quantile-Quantile plot analysis. Outliers were identified using the rstatix package identify_outliers() function. Values that exceed 1.5 times the interquartile range above the third

quantile (Q3 + 1.5xIQR) or 1.5 times the interquartile range below the first quantile (Q1 - 1.5xIQR) are defined as outliers. The Quantile-Quantile plot (**Suppl. Fig. S9G**) also identified the mentioned outliers among the HCs that fell out of the normal distribution. Further analysis was performed with the remaining 44 HCs. More information on the panel and protein-specific validation is also accessible from www.olink.com/downloads. As described in the pre-registration stored on the KI Electronic Notebook, no other proteins in this panel were analysed within this study.

Generation of *Gdnf*^{cHyper} knock-in allele. The targeting construct for the *Gdnf*^{cHyper} allele (**Suppl. Fig. S1A**) contained a 4021 bp 5' homologous arm spanning part of the second intron as well as the entire coding sequence of the mouse *Gdnf* gene, a 610 bp cassette containing the bovine growth hormone polyadenylation signal (bGHpA) in an inverted orientation flanked by the FLEx system (59) starting immediately after the stop codon, a 2615 bp Pgk1-puΔtk-bGHpA sequence flanked by Frt sites, and a 2927 bp 3' homologous arm. The targeting vector was synthesized by GenScript (NJ, USA) and confirmed by restriction analysis and sequencing. G4 embryonic stem (ES, 129S6/C57BL/6Ncr hybrid) cells were electroporated with 30 µg of linearized targeting construct. After puromycin (1.5 µg/ml; Sigma) selection, ES cell colonies were screened by long-range PCR for both 5' and 3' homologous arms and correct PCR products were verified by sequencing. Correctly targeted ES cells were injected into C57BL/N6Crl (Charles River Laboratories, MA, USA) mouse blastocysts to generate chimeric mice. Germline transmission was achieved by breeding male chimeras with C57BL/N6Crl females. The Pgk1-puΔtk-bGHpA sequence was removed using the CAG-Flp mouse line at F2 generation.

Mouse strain maintenance. All mouse strains (Nestin-Cre, Esr1-Cre, Pgk1-Cre and *Gdnf*^{cHyper} mice) were maintained in a 129Ola/ICR/C57bl6 mixed genetic background. Nestin-Cre, Esr1-Cre and Pgk1-Cre strains were maintained as heterozygotes to reduce possible artefacts caused by Cre overexpression. *Gdnf*$^{wt/cHyper}$;Nestin-Cre x *Gdnf*$^{wt/cHyper}$ breedings were used to obtain Nestin-Cre-expressing mice homozygous for *Gdnf*^{cHyper} allele. *Gdnf*$^{wt/cHyper}$;Esr1-Cre x *Gdnf*$^{wt/cHyper}$ breedings were used to obtain Esr1-Cre-expressing mice homozygous for *Gdnf*^{cHyper} allele. Since Pgk1-Cre is ubiquitously expressed and because increased endogenous GDNF

expression in the kidney leads to postnatal lethality (35), we did not attempt to obtain homozygous *Gdnf*$^{cHyper/cHyper}$;Pgk1-Cre mice and instead used heterozygous *Gdnf*$^{wt/cHyper}$;Pgk1-Cre mice for analysis. In these experiments, pups were sacrificed at P3 and possible postnatal lethality due to kidney dysfunction was not evaluated. *Gdnf*$^{wt/cHyper}$ x *Gdnf*$^{wt/cHyper}$ crosses were used for experiments involving striatal AAV-Cre delivery.

AAV production. Pseudotyped AAV2/5 vectors were produced using a double-transfection method with the appropriate transfer plasmid and the helper plasmid containing the essential adenoviral packaging genes, as described previously (146). Vectors were purified by iodixanol step gradients and Sepharose Q column chromatography. The purified viral vector suspension was titrated with TaqMan quantitative PCR and primers targeting the WPRE sequence.

Stereotaxic surgery. 3-5-month-old male mice were anesthetized with isoflurane (3-4% for induction and 1.5-2% for maintenance; Oriola, Finland) in 100% oxygen. The hair on the top of the head was shaved and cleaned with Desinfektol P (Berner Pro, Finland). Mice were placed on a stereotaxic apparatus and lidocaine (Yliopiston Apteekki, Finland) was injected into the skin for local analgesia. The skin was cut with a scalpel and holes were drilled into the skull bilaterally. Two different sets of coordinates were used throughout the study: 1) A/P 1.2, M/L ±2.2 mm and A/P 0.2, M/L ±2.2 mm, or 2) A/P 0.7, M/L ±2.2, relative to the Bregma. With respect to virus diffusion and *Gdnf* expression, both injection regimens produced a comparable outcome. A 33G blunt NanoFil needle (World Precision Instruments, USA) was inserted into the brain parenchyma at a 10-degree angle until reaching D/V -3.0 mm, relative to Bregma. Either 1.0 µl (injections according to the first set of coordinates) or 1.5 µl (injections according to the second set of coordinates) of AAV5-Cre in Dulbecco's PBS, corresponding to 1.7×10^{11} viral genome copies, were injected into each site at a flow rate of 0.2 µl/min. The needle was kept in place for an additional 4-5 minutes and retracted slowly. The skin was closed with sutures and animals were subcutaneously injected with 5 mg/kg of Rimadyl or Norocarp (Yliopiston Apteekki, Finland) in saline for post-operative analgesia. Wild-type littermates were used as controls and were injected with the same dose of AAV5-Cre as the *Gdnf*^{cHyper} mice.

Tamoxifen injection. For the analysis of Gdnf mRNA and protein levels, *Gdnf*$^{wt/cHyper}$ mice were crossed to Esr1-Cre mice and pregnant females were injected i.p. with 25 mg/kg of tamoxifen (Sigma-Aldrich) in corn oil on three consecutive days between E12.5-E14.5. The pups were dissected at P3 and tissues were isolated for further analysis. For RNAscope analysis, pregnant females were injected with 25 mg/kg tamoxifen at E10.5 and tissues were harvested at E14.5. The sex of the pups was not determined.

Prepulse inhibition. The PPI test was performed to evaluate defects in sensorimotor gating, as described previously (48). Male *Gdnf*^{cHyper};Nestin-Cre mice were analysed at an age of 3-4 months by experimenters blind to the genotypes of the mice. Littermates were used as controls. Briefly, mice were placed in a transparent plastic tube (inner diameter 4.5 cm, length 8 cm) that was fixed on a platform inside a startle chamber (Med Associates). Habituation to test environment was performed using white background noise of 65 dB for 5 min. Testing was performed in 12 blocks of 5 trials with five trial types (40-ms, 120 dB startle stimulus (SS) alone, or preceded by a 20-ms prepulse stimulus (PPS) of 68, 72, 76 and 80 dB white noise bursts). The delay between PSS and SS was 100 ms. The first and last block included only SS trials. In blocks 2 through 11, PSS+SS trials were presented in a pseudorandomized order such that each trial type was only presented once within the block. The inter-trial interval was between 10 to 20 ms. The startle response (arbitrary units) was recorded for 65 ms starting with the onset of SS and averaged for each trial type. Startle responses in trials during test phase (blocks 2-11) were averaged for quantification. The prepulse inhibition was calculated using the following formula: 100 − [(startle response on PPS + SS trials/startle response on SS trials) × 100].

T-maze. T-maze was performed on 3-4-month-old male mice. The T-maze was made of grey PVC. Each arm measured 30 cm ×10 cm. A removable central partition extended from the center of the back goal wall of the T to 7 cm into the start arm. This completely prevented the mouse from seeing or smelling the nonchosen arm during the sample run, thus minimizing interfering stimuli. The entrance to each goal arm was fitted with a guillotine door. Each trial consisted of an information-gathering, sample run, followed immediately by a choice run. For the sample run, a mouse was placed in the start arm, facing away from the choice point with the central partition

in place, door on start arm closed and doors to goal arms opened. After opening the door on start arm, the mouse was allowed to choose a goal arm and confined there for 30 s by lowering the respective guillotine door. Then the central partition was removed, the mouse replaced to the start arm (door closed), the doors to both goal arms were opened again and by opening the start door the choice run was started. Alternation was defined as entering the opposite arm to that entered in the sample trial (whole body, including tail). Three trials were run per day with an inter-trial interval at least 30 min, on two consecutive days (6 trials altogether).

Puzzle box. The puzzle box test (147) was performed on 3-4-month-old mice and was devised to demonstrate the motivation of a mouse to solve problems of increasing difficulty when exposed to a brightly lit arena. This test takes advantage of the spontaneous exploratory behaviour of a mouse with the drive to escape an open, brightly lit area. The apparatus was custom-built of white acrylic with dimensions of 45 × 20 × 20 cm. The box was divided into two parts: 2/3, designated as 'start zone', was white and brightly illuminated (~2000 lx, by 2 40W light-bulbs above the compartment), 1/3, designated as "goal zone", was painted black, covered by the lid and separated from the start zone with a partition containing an opening (13 x 5 cm) at the floor level. The opening enabled visibility of and access to the goal zone from the start-zone. The mouse was placed within the start zone and the latency to move into the goal zone via the entrance was recorded over 6 successive trials, which increased in difficulty. Each mouse was taken from its home cage and placed into the start zone facing the end wall parallel to the partition, and the latency to enter into the goal zone, when all four paws were contained, was recorded for each trial. The mouse was returned to for 30 seconds between trials. The trials performed were: Trial 1. Big entrance: goal zone accessible through a big opening. Trial 2. Small entrance: the entrance size was reduced. Trial 3. Paper gate: small entrance blocked with a light paper that mice were able to push to enter the goal zone. Trial 4. Foam plug: small entrance blocked with a piece of foam which was easy to remove by pushing away. Trial 5. Tunnel: access to goal zone only through a transparent acrylic tube of 6 cm that led to the small entrance. Trial 6. Blocked tunnel: about one third of the transparent tube was blocked with bedding material. The block was located closer to the goal zone door entrance, so that the mice were able

to first access to the tunnel and get to the goal zone after digging. The latencies of each trial were recorded for each mouse individually and compared between genotypes.

Marble burying test. The marble burying test was performed to evaluate compulsory behaviour. 3-4-month-old male *Gdnf*^{cHyper};Nestin-Cre mice were placed in a home cage environment containing approximately 5 cm of bedding and 20 marbles in a 4-by-5 array on top of the bedding. The cage lids were closed, and mice were removed after 10 minutes. The number of buried marbles, defined as > 50% of the marble being covered by the bedding material, were counted by an investigator blinded to the genotypes of the mice.

Open field. The open field test was performed on 3-4-month-old male *Gdnf*^{cHyper};Nestin-Cre mice to evaluate general locomotor activity and anxiety-like behaviour. The mice were placed in a novel open field arena (30 × 30 cm, Med Associates) for 30 min and their horizontal and vertical activity were recorded. The peripheral zone was defined as a 6-cm wide corridor along the wall. Distance travelled and time spent in each compartment were recorded by Activity Monitor v5.1 software (Med Associates).

Home cage activity. Spontaneous home cage activity was measured using the InfraMot cages equipped with a heat sensor to register the gross activity of the animals (TSE Systems). Briefly, 3-4-month-old male mice were single housed for 7 days with *ad libitum* access to food and water under a 12/12 h light cycle (lights on at 06:00). Mouse weight, food consumption (via food weight difference), and water consumption (via water weight difference) were measured daily by the experimenter during the second half of the light cycle. The mice were provided with nesting material for enrichment. The first 3 days were considered the habituation period. The activity counts per each circadian hour were recorded by TSE LabMaster V4.2.8 and averaged over a period of last 4 days of monitoring.

IntelliCage. The IntelliCage test (86) was performed on 3-4-month-old female mice to evaluate water consumption, learning and memory, and impulsivity, as previously described (48, 86), with minor modifications. One week before the onset of testing, RFID transponders (Planet ID GmbH, Germany) were subcutaneously injected into the dorso-cervical region under isoflurane inhalation anesthesia. The mice were maintained under a 12/12 h light cycle (lights on at 06:00) at controlled temperature (21 ± 1 °C) and humidity (50–60%) with *ad libitum* access to food.

Apparatus and procedure. The IntelliCage apparatus (TSE, Germany) was placed in a polycarbonate cage (20.5 cm high, 58 × 40 cm top, 55 × 37.5 cm bottom, Tecniplast, 2000P, Buguggiate, Italy). The food was covered with bedding (aspen chips 5 × 5 × 1 mm, Tapvei Oy, Finland) and provided 4 central red shelters (Tecniplast, Buguggiate, Italy). Four triangular conditioning chambers (15 × 15 × 21 cm) were fitted in the cage corners and provided room for one mouse at a time. Each chamber contained two drinking bottles, accessible via two round openings (13 mm diameter) which could be closed by motorized doors. Three multicolor LEDs were mounted above each door and the chamber ceiling contained a motorized valve for the delivery of air puffs. Mice that accessed a chamber were identified by a circular RIFD antenna at its entrance (30 mm inner diameter) and the duration of their visit was determined by both the antenna reading and a temperature sensor that detected the presence of the animal inside the corner. During a visit, the number and duration of individual nosepokes at each door were recorded using IR-beam sensors. Licking episodes at each bottle were monitored using lickometers (duration of the episode, number of licks and total contact time were recorded). Recorded data was analyzed with IntelliCage Plus software.

Habituation. The mice were first habituated to the IntelliCage apparatus and a fixed drinking schedule. During the first 6 days, all doors were open, providing free access to all eight drinking bottles. During the next 3 days, all doors were closed but could be opened once per visit with a nose-poke for 5 s (nose-poke adaptation,). During the next 5 days, the mice were adapted to a fixed drinking schedule with doors opening in response to nose-pokes between the hours of 20:00–22:00 and 04:00–06:00 only.

Flexible Sequencing (FS). During this task, water bottle availability was shuttled between two opposite corners and access to water was available during two 2h sessions per night between the hours of 20:00–22:00 and 04:00–06:00, as during habituation. Water was not accessible in the

remaining two corners. Each mouse could open the gate with a nose poke and drink water for 4s from the active rewarded corner, after which the corner became inactive and the rewarded corner at the opposite end of the apparatus became active. Alternation of corner assignment was controlled for each mouse separately and corner assignments were balanced between mice. The test consisted of four nights (8 drinking sessions) of learning, followed by reversal learning, for a total of four flexible sequencing sessions. The number of visits in each corner were counted by the software and the percentage of correct visits was calculated.

Impulsivity. During the motor impulsivity task, all corners contained bottles with water and operated 24h/day. The first nose-poke during a visit determined the correct side and initiated a delay period (0.5, 1.5, 2.5 or 3.5s, varied randomly), followed by 5 s during which 3 green LEDs above the door on the correct side switched on and the door opened for drinking. A nose-poke during the delay period was considered a premature response, whereas the first nose-poke at the open door was counted as a correct response. Correct response latency was defined as the time that elapsed between the onset of the light stimulus and the correct response.

Isolation of tissues. Pregnant females were deeply anesthetized with CO_2, followed by cervical dislocation and decapitation, and embryos were dissected at indicated time points. P3 mice were sacrificed by decapitation. The kidneys were either immersed in 4% formaldehyde (Sigma, in 1× PBS) overnight, followed by dehydration and paraffinization (Leica ASP 200), or immediately snap-frozen on dry ice and stored at -80 °C until analysed for mRNA and protein expression. The sex of the pups was not determined.

To isolate samples for biochemical analysis, adult mice were deeply anesthetized with CO_2, followed by cervical dislocation and decapitation. The brain was quickly dissected and immersed in ice-cold PBS. Brain regions of interest were isolated from 2-mm slices cut using a brain matrix (Stoelting). Tissue samples dedicated to RNA extraction or HPLC, as well as to kidney GDNF ELISA, were immediately snap-frozen on dry ice and stored at -80 °C until processed. For GDNF ELISA, brain samples were immediately lysed in lysis buffer (see below) and stored at -80 °C until analysis. Males and females were used for qPCR and protein analyses. HPLC and RNAseq were performed on males.

To isolate samples for immunostaining or *in situ* hybridization, adult mice were anesthetized with pentobarbital (Mebunat, 200 mg/kg, i.p., Yliopiston Apteekki) and perfused with warm PBS and 4% formaldehyde (Sigma). Samples were post-fixed in 4% formaldehyde for 24 hours at room temperature and paraffinised or dehydrated in 30% sucrose (Thermo Fisher Scientific) in PBS prior to sectioning. Sucrose-dehydrated tissues were cryosectioned to a thickness of 30 µm and stored in a cyropreservant buffer containing 30% ethylene glycol (Fisher) and 20% glycerol (Acros Organics) in phosphate buffer at -20 °C until analysis.

RNA and protein isolation. Total RNA was isolated using Trizol Reagent (Thermo Fisher Scientific) or RNAqueous Micro Kit (Thermo Fisher Scientific). Tissue samples for protein analysis were homogenized in a lysis buffer prepared according to the recipe in the GDNF Emax Immunoassay System protocol (Promega). The tissue homogenate was centrifuged at 5000 rpm for 15 minutes at 4 °C and the supernatant was either used immediately or stored at -80 °C until further processing. Each protein sample was only thawed and used once.

Genotyping. Genotyping samples were collected at weaning and/or during dissection. Genomic DNA was isolated from the tail using Extracta DNA Prep for PCR - tissue (Quanta Biosciences, USA). Routine genotyping was performed using AccuStart II GelTrack PCR SuperMix (Quanta Biosciences, USA) and analysed by electrophoresis using 1.5-2% agarose gels in borate buffer. The primers used for genotyping the mice for the presence of $Gdnf^{cHyper}$ allele are indicated on **Suppl. Fig. S1A** and **Table S3**.

RNA sequencing. Total RNA was quality checked on an Agilent 2200 TapeStation system. RNA quantity was measured using a NanoDrop ND-1000 Spectrophotometer and 200 ng RNA/sample were used for library preparation using TruSeq Stranded mRNA protocol (Illumina). The yield and quality of the amplified libraries were analysed using Qubit (Thermo Fisher) and the Agilent 2200 TapeStation system. The indexed cDNA libraries were normalised and combined, and the pools were sequenced on the Illumina Nextseq for a 75-cycle v2.5 sequencing run, generating 75 bp single-end reads. Base calling and demultiplexing were

performed using CASAVA software with default settings generating Fastq files for further downstream mapping and analysis.

Fastq files were aligned to the mouse reference genome (mm10) employing the STAR aligner (RRID:SCR_004463) (148). Mapped reads from the subsequent bam files were counted in annotated exons using featureCounts (RRID:SCR_012919) (149). Reference genome sequence in fasta format and entrez gene annotations were retrieved from UCSC. The resulting count table was imported to R/Bioconductor and differential gene expression analysis was carried out using edgeR (RRID:SCR_012802) (150). Analysis was performed on genes that had 1 count per million (cpm) in at least 3 samples and normalised with TMM normalisation followed by trended dispersion estimation. Genes with an FDR adjusted p-value under 0.1 were deemed significant and were used for GO analysis using the topGO library (RRID:SCR_014798) (151). A similarity matrix was constructed of the top 150 GO terms with the GOSemSim library (152), which was then clustered by t-SNE. The t-SNE result was cut into clusters using dynamic tree cutting with the dynamicTreeCut library (153). Plots were prepared with ggplot2 (RRID:SCR_014601) (154). For the construction of the protein-protein association graph, the R igraph library (RRID:SCR_019225) (155) was used with the STRINGdb package (156) to obtain protein-protein interactions from the STRING database. Plotting of the graph was done with the ggraph package (157).

Analysis of protein levels. Quantification of GDNF protein levels in the brain and kidney was performed using GDNF Emax Immunoassay (Promega) with acid treatment. Due to high background in protein samples derived from the striatum, a negative control sample from *Gdnf*$^{cKO/KO}$;Nestin-Cre mice, dissected at the same time as experimental samples and obtained from an animal of comparable age, was always included in GDNF ELISA plate. We were not able to detect a difference between wild-type and *Gdnf*$^{cKO/KO}$;Nestin-Cre samples if the brain tissue was snap-frozen before lysis, therefore fresh tissue was always lysed before freezing and each frozen sample was thawed and used once. 20-100 μg of total protein from the brain or kidney, measured with the DC Protein Assay (Bio-Rad), was loaded onto the ELISA plate. Signal obtained from the *Gdnf*$^{cKO/KO}$;Nestin-Cre negative control sample was subtracted from experimental striatal samples. All samples were analysed in duplicate.

High-performance liquid chromatography. The levels of dopamine and its metabolites were analysed as previously described (158), using HPLC with electrochemical detection.

Histological analysis and *in situ* hybridization. For histological analysis, 5-µm paraffin sections were stained with Harris's hematoxylin (Merck) and eosin Y (Sigma-Aldrich), according to a routine protocol. For fluorescence immunostainings, 30-µm cryosections were permeabilized in PBS with 0.3% TritonX-100 (Fisher Scientific), blocked with 5% normal donkey serum (Abcam), and stained at 1:1000 with a rat anti-DAT primary antibody (Millipore Cat# MAB369, RRID:AB_2190413) followed by an anti-rat AlexaFluor 488-coupled secondary antibody at a concentration of 1:500 (Abcam Cat# ab150153, RRID:AB_2737355). For immunostainings used for stereology, 30-µm free-floating cryosections were quenched with 0.3% H_2O_2 in PBS for 30 minutes, washed trice in PBS, blocked in 0.3% TritonX-100 (Fisher Scientific) with 5% normal horse serum (Abcam) and stained at 1:1000 with a mouse anti-tyrosine hydroxylase (TH) antibody (Sigma-Aldrich, Cat# MAB318, RRID:AB_2313764) followed by a biotinylated anti-mouse antibody (Vector Laboratories Cat# BA-2001, RRID:AB_2336180). Next, slices were stained with diaminobenzidine (DAB) using the Vectastain Elite ABC peroxidase kit (Vector Laboratories Cat# PK-4000) and the Vectastain DAB Substrate peroxidase kit (Vector Laboratories Cat# SK-4100) according to manufacturer's protocols. Slices were then mounted to SuperfrostPlus slides (Thermo Cat# J1800AMNZ), air dried, dehydrated through a series of ethanol (EtOH), and cleared with xylene (2-min 50% EtOH, 2-min 70% EtOH, 3-min 96% EtOH, 3-min 100% EtOH twice, 5-min xylene thrice). Finally, slides were covered with coverslips using Pertex mounting medium (Cellpath, Hemel Hempstead, UK). RNA *in situ* hybridization was performed as previously described (35). Briefly, fresh 5-µm paraffin sections from embryonic kidney or adult brain were hybridized with RNAscope (159) probes (Advanced Cell Diagnostics) detecting Gdnf mRNA, according to the manufacturer's recommendations. Sections were scanned with a Pannoramic 250 digital slide scanner (3D Histech).

Image analysis. Stained mouse prefrontal cortical sections from 4-6 male mice per experimental group of the prelimbic area (approximately 1.78 mm from Bregma, according to the brain atlas

from Paxinos & Franklin, 2001) and frontal association cortex (approximately 2.68 mm from Bregma, according to the brain atlas from Paxinos & Franklin, 2001) were imaged at 20x and 63x magnification using a Zeiss Axio Imager microscope outfitted with a Zeiss ApoTome for optical sectioning. Five successive fluorescence images were taken every 0.5μm using the ApoTome, optically sectioned with structured illumination to produce a 10-12-μm thick 3-D final image. 2-3 images per area were analysed using Imaris software to quantify the density of the stained protein of interest (calculated as percent of tissue defined as total volume of protein stain/total volume of image) by a researcher blind to experimental conditions.

Stereological quantification. Unbiased stereological cell counts of TH+ cell bodies in the substantia nigra pars compacta (SNpc) and ventral tegmental area (VTA) were performed by an experimenter blind to the experimental conditions, as done previously (124). Briefly, every sixth stained 30-μm thick serial cryosections containing the SNpc or VTA, corresponding to approximately -3.08 mm to -3.62 mm from Bregma, according to the brain atlas from Paxinos & Franklin, 2001, were selected. Four SNpc sections and three VTA sections per animal were used. The StereoInvestigator software (MBF Bioscience) was used to carry out the stereological quantification according to the optical dissector workflow (160). For the SNpc, a sampling grid was set up to cover 30% of the area of interest with a counting frame size of 40-μm by 40-μm. For the VTA, the same size counting frame was used with a sampling grid covering 15% of the area of interest. Counting frame positions were randomized by the software for a systematic random sampling of the area of interest. The Gunderson coefficient of error was used to estimate precision and values ≤0.1 were accepted.

Fast-scan cyclic voltammetry. At 6-8 months of age, male mice were decapitated, the brains were removed, and 300-μm-thick coronal slices containing the striatum were cut on a model 7000 smz-2 vibratome (Campden Instruments) in oxygen-bubbled (95% O_2, 5% CO_2) ice-cold cutting artificial cerebrospinal fluid (ACSF) containing the following (in mM): 75 NaCl, 2.5 KCl, 26 $NaHCO_3$, 1.25 NaH_2PO_4, 2 $MgCl_2$, 0.7 $CaCl_2$, and 100 glucose. The slices were allowed to recover in a holding chamber for 20 min at 34°C, followed by a 1-3-hour period at room temperature in oxygen-bubbled (95% O_2, 5% CO_2) recording ACSF containing the

following (in mM): 119 NaCl, 3 KCl, 26 MgSO4, 1 KH2PO4, 1.2 MgCl2, 2 CaCl2, and 10 glucose. In the recording chamber, the slices were perfused continuously with oxygen-bubbled recording ACSF. Fast-scan cyclic voltammetry recordings were performed using cylindrical 5 μm carbon fiber electrodes positioned at the dSTR or VTA ∼50 μm below the exposed surface. Using the medial lemniscus and cerebral peduncle as landmarks, only VTA slices from -3.40 to -3.80 mm from Bregma according to the brain atlas from Paxinos & Franklin, 2001, were selected, as these are the slices that contain the paranigral nucleus. The electrode was placed medial to the medial lemniscus and the FSCV background-subtracted voltammogram was used to identify dopamine, ensuring proper placement of the electrode within the paranigral nucleus. Recordings which contained a reduction peak at approximately -200mV, indicating the presence of serotonin, were rejected. Striatal or VTA slices were stimulated electrically at 2-minute intervals using a stainless-steel bipolar electrode placed ∼100 μm from the recording electrode. For the dorsal striatum, a single electrical square pulse of either 0.4 ms (AAV-Cre injected mice) or 1 ms ($Gdnf^{cHyper}$;Nestin-Cre mice) in duration were produced using an Iso-Flex stimulus isolator triggered by a Master-8 pulse generator (A.M.P.I.). A stimulus magnitude of 200 µA was obtained by selecting the minimum value that produced the maximum response reliably. Triangular voltage ramps from a holding potential of −450 mV to +800 mV over 9 ms (scan rate of 300 mV/ms) were applied to the carbon fiber electrode at 100 ms intervals. In the burst stimulus experiments, a burst of 5 pulses at 20 Hz was used. For the VTA recordings, a burst of 30 pulses at 30 Hz at 2-minute intervals was used to elicit DA release, as described previously (161). The current was recorded using an Axopatch 200B amplifier (Molecular Devices), filtered through a 5 kHz low-pass Bessel filter, and digitized at 40 kHz (ITC-18 board; InstruTech). Triangular wave generation and data acquisition were controlled and the recorded transients were characterized using a computer routine in IGOR Pro (WaveMetrics) (162, 163). Background-subtracted cyclic voltammograms obtained with 1 μM of dopamine solution (dopamine-HCl; Sigma-Aldrich) or 1 µM serotonin solution (serotonin-HCl; Sigma-Aldrich) were used to calibrate the electrodes. For recordings with alpha-methyl-para-tyrosine (AMPT), a TH inhibitor, recordings were done in the dSTR as above; however, after 4 single-pulses (490 seconds), 100µM AMPT was applied to the slice via perfusion in the recording ACSF, and slices were then stimulated as AMPT flowed into the recording chamber.

Istradefylline treatment. Adult male *Gdnf*$^{cHyper/cHyper}$;Nestin-Cre and *Gdnf*$^{wt/wt}$;Nestin-Cre mice were single-housed in cages equipped with the activity monitor system InfraMot (TSE Systems) and were allowed *ad libitum* access to water and food, with temperature and humidity held constant. Water intake, food intake, and weight were monitored daily. As described previously (164), prior to beginning istradefylline treatment, instead of water, the mice were given a vehicle solution without istradefylline containing DMSO (Fisher Scientific), Kolliphor EL (Sigma), and Light Mineral Oil (Sigma) with 0.3% saccharine (Sigma) for 3 days. On the final day of vehicle solution administration, the drinking baseline according to the amount each individual mouse had consumed was used to calculate the approximate concentration of istradefylline to be added to the vehicle solution such that each mouse was expected to consume approximately 4 mg/kg/day of istradefylline (MedChemExpress, Cat# HY-10888). The amount of consumed istradefylline solution was measured daily throughout the experiment and istradefylline concentration in the drinking solution was adjusted to achieve a sustained constant daily dose of approximately 4 mg/kg/day (**Suppl. Fig. S8A-B**). After 10 days, mice were switched back to the vehicle solution without the drug for 3 days before they were sacrificed, and relevant tissues were isolated.

Quantification and Statistical Analyses

Sample size. In experiments involving mice, sample size was determined on the basis of previous studies using mice with the same triple-mixed genetic background. No statistical methods were used to predetermine sample size.

Rules for stopping data collection. Rules for stopping data collection were not defined.

Data inclusion/exclusion criteria and handling of outliers. Data was excluded based on insufficient quality (assessed immediately after acquisition and before analysis). Quality control parameters, where relevant, are detailed in the corresponding Methods sections above. Grubbs test for outliers were performed when outliers were suspected, and significant outliers ($p<0.05$) were excluded from downstream analysis.

Research subjects or units of investigation. In human studies, research subjects correspond to individual FEP patients or healthy controls. In animal experiments, research subjects correspond

to individual animals, with the exception of fast scan cyclic voltammetry experiments, where the experimental units are brain slices. Please see the corresponding Methods sections for further details.

Randomization. Because the genotype of the animal determined the experimental group of the animal, randomization to treatment was not possible. To reduce possible bias due to environmental variables, the following criteria were applied. In animal experiments, only litters including animals from different genotypes were included. In behavioural tests, animals were tested in a random order. AAV-Cre injections were administered to all experimental animals (wild-type and conditional hypermorph) in a random order. Tissue isolation was always performed in a random order.

Blinding. In experiments involving embryonic or postnatal *Gdnf*^{cHyper} animals, the samples were processed before genotyping. Tissue samples from adult animals were collected and analysed by investigators blinded to experimental groups. Behavioural tests were performed by investigators blinded to experimental groups. In cyclic voltammetry experiments on AAV-Cre-injected mice, two mice from different genotypes were analysed each day. In cyclic voltammetry experiments in Nestin-Cre mice, sections from one mouse were analysed each day. In both cases, the order of animals was assigned randomly by a second investigator not involved in data acquisition and the investigator performing the experiment was blinded to the genotypes of the animals.

Statistical analysis. All values are presented as mean ± standard error of the mean. Statistical comparison between two groups was performed using an unpaired Student's *t*-test or Welch’s t-test, where appropriate. Multiple comparisons were performed with one-way or two-way analysis of variance (ANOVA), followed by Tukey's or Sidak’s *post hoc* test. Correlation analyses were performed using Pearson product-moment correlation. P-values were adjusted for multiple testing using the Bonferroni correction as indicated. Statistical analysis was performed with GraphPad Prism v8 and ggplot2. Quantitative PCR data was analysed as described previously (165), using the geometric mean of reference genes for normalisation. The level of statistical significance was set at $P < 0.05$.
